# Decreased function of fatty acid desaturase 1 is associated with placental foetal vascular malperfusion and highlights the key role of the maternal–foetal interface

**DOI:** 10.1016/j.ebiom.2026.106517

**Published:** 2026-10-17

**Authors:** Zhenjie Liu, Ze Long, Stephanie Nartey, Li Tao, Shahrazad Saab, Robert Ty Partridge, Carolyn M. Salafia, Zheyun Peng, Yang Jiang, Tengfei Ma, Douglas Ruden, Nigel Paneth, Jean M. Kerver, Raina Nakova Fichorova, Wanqing Liu

**Affiliations:** a Department of Pharmaceutical Sciences, Eugene Applebaum College of Pharmacy and Health Sciences, Wayne State University, Detroit, MI, 48201, USA; b Department of Orthopedics, The Second Xiangya Hospital of Central South University, Changsha, China; c Laboratory of Genital Tract Biology, Department of Obstetrics, Gynecology and Reproductive Biology, Harvard Medical School, Brigham and Women's Hospital, Boston, MA, USA; d Department of Physiology, Wayne State University School of Medicine, Detroit, MI, USA; e Department of Pathology, University Hospitals Cleveland Medical Center, Case Western Reserve University, Cleveland, OH, USA; f Department of Psychology, Wayne State University, Detroit, MI, USA; g Placental Analytics LLC, New Rochelle, NY, USA; h Institute for Basic Research in Developmental Disabilities, Staten Island, NY, USA; i Department of Public Health Sciences, Henry Ford Health, Detroit, MI, USA; j Department of Nutrition and Health Science, Ball State University, Muncie, IN, USA; k Department of Obstetrics and Gynecology and the Institute of Environmental Health Sciences (IEHS), Wayne State University, Detroit, MI, USA; l Departments of Epidemiology & Biostatistics and Pediatrics and Human Development, College of Human Medicine, Michigan State University, East Lansing, MI, USA; m Department of Epidemiology and Biostatistics, College of Human Medicine, Traverse City Campus, Michigan State University, Traverse City, MI, USA; n Department of Pharmacology, School of Medicine, Wayne State University, Detroit, MI, USA; o Department of Oncology, School of Medicine, Wayne State University, Karmanos Cancer Institute, Detroit, MI, USA

**Keywords:** Polyunsaturated fatty acids, FADS1, Placental foetal vascular malperfusion, Polymorphism, Maternal-foetal interface

## Abstract

**Background:**

Placental foetal vascular malperfusion (PFVM) is a chronic disorder characterised by impaired blood flow and abnormal vascular development, inducing adverse neonatal outcomes. Long chain polyunsaturated fatty acids (LC-PUFAs) play a crucial role in placenta health. We aimed to explore the role of genetic variation in LC-PUFA metabolism in PFVM risk as well as underlying mechanisms.

**Methods:**

Associations were examined between two functional polymorphisms (rs174538 and rs174556) of the fatty acid desaturase 1 (*FADS1*) gene encoding the rate-limiting enzyme for LC-PUFA metabolism and PFVM among pregnant women (n = 646). Placental transcriptomes were correlated with PFVM and *FADS1* genotypes. Various assays were performed in human umbilical vein endothelial cells and trophoblasts with *FADS1* inhibitor and LC-PUFA interventions to characterise their function changes.

**Findings:**

Low-function alleles of both polymorphisms were significantly associated with increased PFVM risk and decreased placental *FADS1* transcription level. In placenta tissue, lower *FADS1* expression is also associated with PFVM. PFVM-associated genes are enriched to pathways related to immune responses, apoptosis, and angiogenesis, which overlap with the genes and pathways associated with the risk alleles and demonstrate a cell-type enrichment to endothelial cells. In vitro, FADS1 inhibition impairs multiple functions of and interactions between endothelial and trophoblast cells. These impairments are reversed by LC-PUFA supplementation.

**Interpretation:**

These findings highlight the impact of maternal LC-PUFA homoeostasis in placenta health and suggest that maternal *FADS1* variants can potentially be an early diagnostic marker for PFVM. Our data supports PUFA metabolism as a precision therapeutic or preventive target to reduce placental dysfunction risk.

**Funding:**

10.13039/100000052NIH, 10.13039/100006710Wayne State University and 10.13039/100006534Michigan State University.

**Research in context**

**Evidence before this study**

Prior to conducting this study, we reviewed a broad spectrum of research on placental pathologies, lipid metabolism, and pregnancy complications, drawing from PubMed, Embase, and Web of Science. The search focused on studies from 2019 to 2026, using keywords “placental vascular pathology,” “PUFA desaturation,” “*FADS1*,” “transcriptome,” “polyunsaturated fatty acids,” and “pregnancy complications.” Both clinical and preclinical studies were considered, without restriction on language. Inclusion criteria required studies that explored the role of fatty acids and desaturation enzymes, particularly *FADS1*, in pregnancy-related pathologies. Quality assessments for included studies were conducted using established risk-of-bias frameworks, evaluating the methodological rigour of each study. Evidence indicated that *FADS1* genotype is a key factor influencing maternal circulating PUFA levels. Also, the maternal systemic PUFA level exerts a significant impact on placental and foetal PUFA profile. In addition, dysregulation of PUFA pathways contributes to placenta-related perturbations. However, there was a lack of studies performed to investigate the role of PUFA metabolism, FADS1, or its genetic variations in placental foetal vessel pathologies prior to this study.

**Added value of this study**

The early diagnosis of PFVM is not available and molecular mechanism underlying PFVM remains elusive. This study advances the current understanding of how reduced PUFA desaturation, driven by maternal low-function *FADS1* alleles, impacts placental health. Specifically, our study demonstrates for the first time that decreased maternal *FADS1* activity is associated with PFVM. Mechanistically, the maternal *FADS1* genotype is associated with placental *FADS1* expression, while lower placental expression of *FADS1* is also associated with PFVM. Placental transcriptomic pathways associated with PFVM and those associated with *FADS1* genotypes are commonly shared. While FADS1 is primarily expressed in foetal trophoblasts and endothelial cells, reduced *FADS1* activity or expression in these cells is associated with impaired cellular functions and interactions between the two cell types, which are reversed by the supplementation of *FADS1* LC-PUFA products. This research for the first time links maternal genetic variation in PUFA metabolism to placental pathologies and provides new potential interventional targets to improve maternal and foetal health during pregnancy.

**Implications of all the available evidence**

The findings of this study, coupled with the existing body of literature, highlighted the key role of the maternal LC-PUFA homoeostasis in influencing placental health. Furthermore, the maternal genetic variation in PUFA metabolism is associated with increased susceptibility to placental pathologies, suggesting that monitoring maternal *FADS1* genotype and PUFA desaturation activity in pregnant women could potentially provide early indications of placental pathologies. Moreover, our study rationalises future research directions, e.g. dietary interventions or supplementation with long-chain PUFAs could potentially mitigate the risk of developing conditions such as PFVM, particularly among patients carrying the risk alleles. These results highlight the need to further investigate the molecular mechanisms of PUFA metabolism in the placenta and explore precision preventive and therapeutic strategies to promote placenta and neonatal health.

## Introduction

Placental foetal vascular malperfusion (PFVM) is a chronic disorder that disrupts blood vessels in the placenta, reducing blood flow to the developing foetus.^1^^,^^2^ This impairment affects the exchange of essential nutrients and oxygen at the maternal–foetal interface, often leading to adverse pregnancy outcomes such as intrauterine growth restriction (IUGR), preterm labour and birth, stillbirth, and neurological impairments in newborns.^3^, ^4^, ^5^ In the United States, approximately 10% of pregnancies result in preterm birth (less than 37 weeks).^6^ According to the Commonwealth Fund the incidence of severe pregnancy outcomes has escalated, with over 50,000 women annually experiencing serious childbirth-related complications.^7^, ^8^, ^9^, ^10^ The main pathophysiology of PFVM includes placental vascular constriction or thrombosis, reduced blood vessel formation and angiogenesis, placental infarcts, umbilical cord abnormalities, and abnormal inflammatory processes.^1^^,^^11^, ^12^, ^13^, ^14^, ^15^, ^16^, ^17^, ^18^ Pathologically, PFVM is characterised into different foetal vascular pathologies (FVPs), such as avascular villi (AVV), stromal karyorrhexis (SK), hemorrhagic endovasculitis (HEV), foetal stem mural thrombosis (FTHR), chorionic plate vessel mural thrombosis (CPTHR), and among others.^1^

Various factors, including genetic, environmental, and hormonal influences, may predispose individuals to PFVM.^1^^,^^2^^,^^17^^,^^19^, ^20^, ^21^, ^22^ For example, the deficiency of epidermal growth factor-like domain 7 (EGFL7), which is a secreted angiogenic factor in endothelial cells, was shown to lead to placental malperfusion in mouse.^19^ Maternal smoking or exposure to chemical or air pollution can disrupt placental development^23^^,^^24^ and lead to PFVM. Maternal gestational diabetes and disorders of carbohydrate metabolism induce the damage of foetal vessels and associated with PFVM.^21^^,^^22^

Polyunsaturated fatty acids (PUFAs) are not only important lipids integral to cell membranes, but they also play crucial roles in cellular signalling, inflammation modulation, and gene expression, supporting foetal development and maternal health.^25^, ^26^, ^27^, ^28^, ^29^, ^30^ PUFAs, especially n-6 and n-3 PUFAs are critically involved in pregnancy complications and promoting healthy outcomes.^31^, ^32^, ^33^, ^34^, ^35^ More specifically, n-6 PUFAs, particularly arachidonic acid (C20:4n-6, ARA) as well as its metabolic derivatives e.g. prostaglandins (such as PGE2s) and thromboxane (such as TXA2), act as pro-inflammatory factors that promote vasoconstriction and platelet aggregation, both of which are associated with foetal stress and pregnancy complications.^36^, ^37^, ^38^, ^39^, ^40^ Other lipid mediators, e.g. lipoxin A, eicosanoids, and docosanoids derived from ARA regulate apoptosis and cellular differentiation,^41^, ^42^, ^43^ essential processes for the remodelling of maternal and foetal arteries during pregnancy. An abnormal abundance or function of these n-6 PUFAs, such as their excessive production, can impair placental function and lead to pregnancy complications, such as preterm labour and preeclampsia.^44^, ^45^, ^46^ On the other hand, n-3 PUFAs, such as eicosapentaenoic acid (C20:5n-3, EPA) and docosahexaenoic acid (C22:6n-3, DHA), as well as their metabolic derivatives e.g. PGE3s, resolvins, maresins etc. can reduce inflammation in the placenta by competing with n-6 PUFAs derivatives.^47^, ^48^, ^49^ n-3 PUFAs and their derivatives not only enhance trophoblast function by promoting VEGF secretion, reducing inflammation, improving nutrient transport, and supporting placental development,^50^^,^^51^ but also enhance the function of endothelial cells,^52^ which line foetal blood vessels, improving foetal oxygenation and nutrient delivery by supporting angiogenesis and promoting vasodilation and blood flow.^53^ Furthermore, n-3 PUFAs reduce the production of thromboxane A2, preventing abnormal foetal vessel thrombosis, distress, and malperfusion.^54^^,^^55^ Balancing the activity and production of n-6 and n-3 PUFAs is critical for ensuring healthy placental vascular function.^33^^,^^34^^,^^56^^,^^57^

However, the variation in PUFA metabolism on PFVM and subsequent pregnancy outcomes remain underexplored. In humans, the bioproduction of long-chain PUFAs i.e. ARA, EPA, and DHA starts from dietary intake of n-3 and n-6 PUFA precursors linoleic acid (LA, C18:2, n-6) and α-linolenic acid (ALA, C18:3, n-3), respectively, by competing the same set of enzymes including desaturases (fatty acids desaturases, more specifically *FADS1* and *FADS2*) and elongases (ELOVs) (Fig. 1). The maternal liver is the primary site of systemic LC-PUFA (PUFAs containing 20 or more carbon atoms) biosynthesis.^58^, ^59^, ^60^ During the pregnancy, the foetal circulating LC-PUFA levels are primarily controlled by the LC-PUFA profile of the maternal system.^61^^,^^62^ Meanwhile, the placenta may also contribute to fatty acid metabolism and regulate the intrauterine transfer and local availability of LC-PUFAs at the maternal–foetal interface during pregnancy. The efficiency of LC-PUFA synthesis is further influenced by multiple nutritional and physiological factors, including dietary intake of LA and ALA, genetic variation in FADS and elongase genes, maternal metabolic status, and pregnancy-related adaptations.^63^^,^^64^ It has been well-established that multiple genetic variants in the *FADS1-FADS2* locus are correlated with expression levels of both genes, especially *FADS1*, and further associated with blood and tissue levels of LC-PUFAs, including both ARA and EPA among general populations,^65^, ^66^, ^67^, ^68^ as well as pregnant women.^69^, ^70^, ^71^, ^72^, ^73^, ^74^ These single nucleotide polymorphisms (SNPs) have been also observed to be associated with multiple placental and perinatological conditions.^27^^,^^75^^,^^76^ However, whether and how these SNPs influence the risk of placental vessel pathologies and pregnancy complications, such as PFVM remains unchecked. In this study, we aimed to: (1) evaluate the association between maternal low-function *FADS1* alleles and the risk of PFVM; (2) characterise PFVM- and *FADS1* genotype–associated placental transcriptomic signatures to identify dysregulated pathways and enriched cell types potentially involved in disease pathogenesis; and (3) functionally validate the impact of altered FADS1 activity as well as supplementation of LC-PUFAs on trophoblast cell and endothelial cell function and behaviour.

**Fig. 1:**
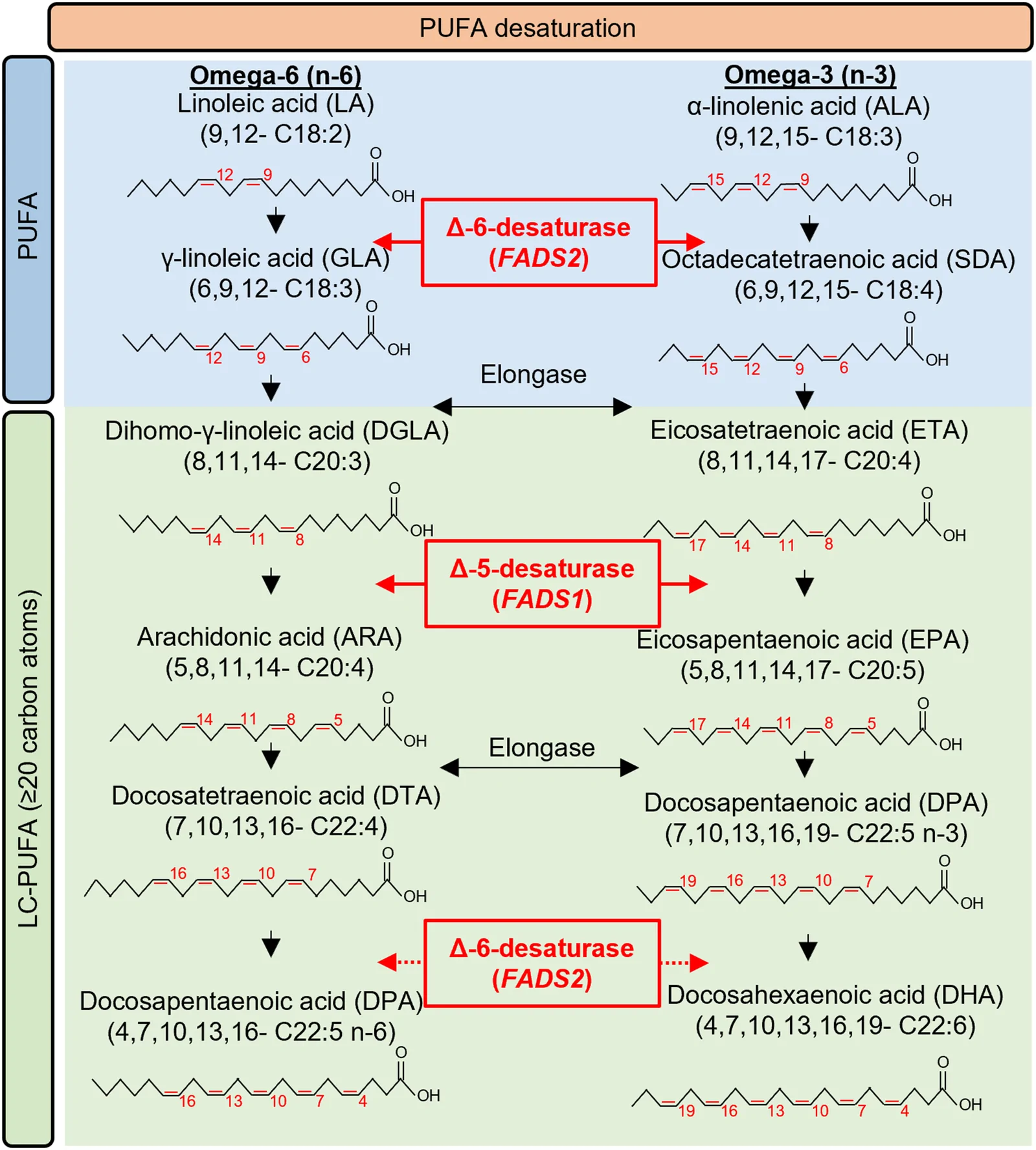
**PUFA desaturation by desaturase.** The diagram shows the n-6 and n-3 PUFA desaturation process. In the n-6 PUFA process, linoleic acid (LA) is converted to γ-linolenic acid (γ-LA) by Δ6-desaturase (encoded by *FADS2*). γ-LA is elongated to dihomo-γ-linolenic acid (DGLA) and further converted to arachidonic acid (ARA) by Δ5-desaturase (encoded by *FADS1*). ARA is subsequently elongated and desaturated to produce docosatetraenoic acid and docosapentaenoic acid (DPA). In the n-3 PUFA process, α-linolenic acid (ALA) is converted to octadecatetraenoic acid by Δ6-desaturase (*FADS2*) and further elongated to eicosatetraenoic acid and further converted to eicosapentaenoic acid (EPA) by Δ5-desaturase (*FADS1*). EPA is then elongated and desaturated to docosahexaenoic acid (DHA).

## Methods

### Ethics

This study uses blood and placenta samples as well as various data previously collected in the project of Michigan Archive for Research on Child Health (MARCH),^77^ based on the established protocol which is fully reviewed and approved by the Michigan State University Institutional Review Board (IRB) [LEGACY16-1429M: 16-1429: Prenatal Exposures and Child Health Outcomes: A Statewide Study (CGA# 149003, 151506); Initial approval: 5/26/2017]. The overarching aim of the MARCH cohort is to understand the impacts of prenatal exposures on maternal and child health outcomes. MARCH cohort was established using a stratified random sampling design of births across Michigan's lower peninsula. Stratification was performed by geographic region and delivery hospital to ensure broad population representativeness. Within each stratum, eligible births were randomly selected for enrolment according to the MARCH study protocol. Participants were enrolled at their first prenatal care appointment at one of 21 prenatal care clinics, delivering at 11 hospitals across the state from 2017 to 2023. After obtaining written informed consent, survey data was collected at three timepoints during pregnancy. Blood samples were collected at the clinic, coordinated with routine clinical care during the first and third trimesters. Hospital staff assisted in collecting participants' placentae at childbirth.

The current study utilised placental and maternal samples obtained from the existing MARCH cohort; no additional stratification or random sampling procedures were applied during sample selection for the present analyses. All samples and data were de-identified prior to arriving at the research lab. The Wayne State University IRB determined this study as “Not Human Participant Research”, and therefore an IRB review is not required (Protocol: WSU-IRB-201968; approval date: July 11, 2019) before the experiments were started.

### Pathological review and assessment for PFVM

Two full thickness samples of the chorionic disk were obtained from each quadrant of the disk at mid position between the disk center and the margin. Each sample was placed in a cassette, dehydrated, and embedded in paraffin. The 5-micron section was stained with haematoxylin and eosin and examined by a single reviewer (CMS) blinded to gestational age. Lesions were scored as binary (present/absent) using definitions for foetal vascular obstructive lesions as provided.^78^^,^^79^

### Selection of representative SNPs

We selected two representative SNPs, rs174538 and rs174556 from the *FADS1-FADS2* locus. Both genes encode rate-limiting enzymes controlling the production of ARA and EPA. Both SNPs have been well documented to be associated with *FADS1* gene expression in the liver (the major organ for LC-PUFA biosynthesis and metabolism) and other human tissues in our previous study^59^ and the GTEx database (GTEx Analysis Release V10) (Supplementary Figure S1A). Both SNPs have been broadly associated with circulation levels of AA, EPA, and DHA among regular populations and pregnant women in previous studies.^69^, ^70^, ^71^, ^72^, ^73^, ^74^

### gDNA extraction and genotyping

gDNA was extracted from maternal blood clot samples using the Quick-DNA 96 kit (ZYMO Research, Irvine, CA, USA). Genotyping of the two SNPs was based on the TaqMan SNP genotyping assays according to the manufacturer's protocol using qPCR in an Applied Systems Viia-7 system (Thermo Fisher Scientific, Waltham, MA, USA).

### FADS1 SNP genotype coding and justification

For genetic association analyses, FADS1-linked single nucleotide polymorphisms (SNPs) rs174538 and rs174556 were analysed under an additive genetic model. Genotypes were coded as ordinal variables (2 = homozygous major allele [wild-type], 1 = heterozygous, and 0 = homozygous minor allele). This coding scheme was selected based on extensive prior evidence demonstrating that the minor alleles of rs174538 and rs174556 are associated with reduced FADS1 expression (Supplementary Figure S1A) and lower circulating levels of LC-PUFAs, including ARA, EPA, and DHA.^68^^,^^80^ Treating genotype as a quantitative variable allows evaluation of allele-dose–dependent effects on placental vascular pathology and transcriptomic outcomes while maximising statistical power. All primary association analyses using this coding were conducted with multiple logistic or linear regression models adjusting for maternal body mass index (BMI), age, race, and gestational age (GA).

### mRNA transcriptome array for placenta samples

An mRNA transcriptome array of 2559 genes from High Throughput Genomics (HTG, Tucson, AZ, USA) and the HTG EdgeSeq host software (version 6.1) were used to profile the gene expression of 102 placentas paired with the blood specimens assessed for PUFA genotyping. The HTG platform utilises the nuclease protection assay (qNPA) previously described and validated by RT-PCR. The array consisted of 2559 nuclease protection probes, including genes specifically selected to represent the know immunoinflammatory cascades, 15 housekeeping genes, five negative control plant gene probes, and spike-in positive control probes, including 10 RNA control ratio mixtures (ER-013, ER-028, ER-067, ER-099, ER-109, ER-120, ER-147, ER-154, ER-160, and ER-171), developed by the External RNA Controls Consortium (ERCC). The placental tissues were fixed in formalin and embedded in paraffin using histopathologic procedures standard for the clinical practice as described. A series of 5-mm thick sections were cut for gene expression and parallel haematoxylin-eosin staining to determine the surface area of the adjacent tissue section needed to calculate volume of the HTG buffer used to lyse the tissue for each qNPA at 3.5 μL buffer/mm^2^ tissue area. The minimum tissue area used in the study was 124 mm^2^. After adding the lysis buffer in the tissue section, the sample was overlayed with 500 μL denaturation oil, centrifuged at 10,000 g and incubated at 95 °C for 20 min. After colling for 10 min, proteinase K was added and the sample for incubated at 50 °C for 3 h followed with processing on the HTG EdgeSeq instrument following manufacturer instructions. The post EdgeSeq processing included PCR adding adaptors and molecular barcodes, purification, qPCR and library preparation for sequencing by Illumina NextSeq instrument and Nextseq 500/550 high output kit v2.5 75 cycles. The Illumina FASTQ files were parsed through the HTG software generating quantitative expression values for each gene in the panel.

### Cell invasion or migration assay

Equal amounts of HTR-8 cells (ATCC; CRL-3271; RRID: CVCL_7162; Manassas, VA, USA) or HUVECs (mixed cells from Lonza: C2519A and Thermo Fisher Scientific: C0035C) were seeded and cultured with LC-PUFAs (4–20 μM) &/or D5D-IN-326 (2 μM; *FADS1* inhibitor^81^) on the insert part of a transwell plate (Thermo Fisher, 8 μm pore size. For invasion assay, Matrigel (Fisher Scientific, Hampton, NH, USA) was used to coat the transwell plate for HTR-8 cells invasion). After 24 h, cotton sticks were used to remove the cells remaining on the upper surface of the inserts. The inserts containing the migrated cells attached to the backside were fixed with 4% paraformaldehyde (Fisher Scientific, Hampton, NH, USA) for 10 min at room temperature. Then, cell nuclei were labelled with 4′,6-diamidino-2-phenylindole (DAPI; Thermo Fisher Scientific, Waltham, MA, USA). All samples were observed and imaged using a Keyence microscope (Keyence, IL, USA).

### Tube formation assay

200 μL of growth factor-reduced Matrigel (Fisher Scientific, Hampton, NH, USA) was coated onto a 12-well plate (Fisher Scientific, Hampton, NH, USA). HUVECs treated with LC-PUFA &/or D5D-IN-326 or co-cultured HUVECs were cultured on the coated plates for 24 h. The conditions of all HUVECs were imaged at 24 h using a Keyence microscope (Keyence, IL, USA). Tube formation was analysed using the angiogenesis analyser tool in ImageJ (version 2024 1.54k, NIH, USA), with node number used as indicators of angiogenesis.

### Cell proliferation and viability evaluation

For proliferation evaluation, all the HTR-8 cells and HUVECs were treated, fixed, and stained with EdU staining kit (Abcam, Cambridge, UK). To assess viability, all cells were incubated with a live/dead cell viability dye (Thermo Fisher Scientific, Waltham, MA, USA) for 20 min. Cell nuclei were stained with Hoechst 33342 (Fisher Scientific, Hampton, NH, USA), and fluorescent images were captured using a Keyence microscope (Keyence, IL, USA).

### *FADS1* gene knockdown

HTR-8 cells were seeded and transfected via specific shRNA lentivirus particles (sh-scramble, sh-*FADS1*; Santa Cruz Biotechnology, Dallas, TX, USA; detail sequence information of each shRNA was shown in the Supplementary Table S1) followed with 5 μg/mL polybrene treatment (Santa Cruz Biotechnology, Dallas, TX, USA). Cells were selected by 10 μg/mL puromycin dihydrochloride (Santa Cruz Biotechnology, Dallas, TX, USA). The condition of the protein knockdown was evaluated by western blot assay.

### Western blot assay

shRNA-transduced cells (sh-scramble or sh-*FADS1*) were cultured in 6-well plates for 48 h and lysed in RIPA buffer (Thermo Fisher Scientific, Waltham, MA, USA) containing a complete protease inhibitor cocktail for 30 min at 4 °C. Lysates were centrifuged at 12,000×*g* for 10 min, and protein concentrations were determined using the Pierce BCA Protein Assay Kit (Fisher Scientific, Hampton, NH, USA). Equal amounts of protein (20 μg) were separated on 4–12% SDS-PAGE gradient gels (Thermo Fisher Scientific, Waltham, MA, USA) and transferred to nitrocellulose membranes (Thermo Fisher Scientific, Waltham, MA, USA). Membranes were blocked with 5% non-fat milk in TBST (Fisher Scientific, Hampton, NH, USA) for 30 min at room temperature, followed by overnight incubation at 4 °C with primary *FADS1* antibodies (Abcam, Cambridge, MA, USA). After washing with TBST, membranes were incubated with the secondary antibodies for 1 h at room temperature. Protein bands were detected using the ChemiGlow West Chemiluminescence Substrate Kit (Thermo Fisher Scientific, Waltham, MA, USA) and imaged with a ChemiDoc imaging system (version 6.0). Band intensities were quantified using ImageJ (NIH, Bethesda, MD, USA)^82^ and normalised to Vinculin.

### Co-culture assay

Equal amounts of sh-scramble or sh-*FADS1* HTR-8 cells were seeded and cultured on the insert part of a transwell plate (0.4 μm pore size; Thermo Fisher Scientific, Waltham, MA, USA), while equal amounts of HUVEC were seeded and co-cultured on the plate bottom. After 24 h, the culture medium was collected for the growth factor release assay. The tube formation evaluation was performed in the HUVECs.

### RNA extraction and RT-qPCR

The total RNA of placenta tissues (FFPE) and HTR-8 gene edited cells was extracted using the Quick-RNA FFPE Miniprep (ZYMO Research, Irvine, CA, USA) or RNA mini kit (Qiagen, Hilden, Germany). RNA concentrations were measured using Nanodrop (Thermo Fisher Scientific, Waltham, MA, USA) to assess purity and yield. cDNA was synthesised using the reverse transcription kit (Qiagen, Hilden, Germany) and quantitative RT-PCR was performed using *FADS1*-specific primers (Supplementary Table S2) and SYBR Green master mix (Thermo Fisher Scientific, Waltham, MA, USA). Negative delta cycle threshold (-Δ Ct) values were calculated by using the housekeeping gene (PPIA).

### Reagent validation and quality control

HTR-8/SVneo cells were obtained from ATCC (CRL-3271; RRID: CVCL_7162), maintained at low passage, routinely monitored for morphology, and periodically confirmed to be mycoplasma-free using PCR-based testing. Primary HUVECs were obtained from Lonza and Thermo Fisher Scientific and used at early passages (less than 10). For Western blotting, the *FADS1* antibody (Abcam- AB126706; RRID: AB_11130088) was previously validated by shRNA-mediated FADS1 depletion, which markedly reduced the corresponding protein signal.^83^ Vinculin (Cell Signalling Technology, 13901; RRID: AB_2728768) was used as a loading control, and HRP-linked anti-rabbit IgG (Cell Signalling Technology, 074; RRID: AB_2099233) was used as the secondary antibody. We also evaluated two FADS1 antibodies, Abcam ab126706 (RRID: AB_11130088) and Sigma–Aldrich HPA042705 (RRID: AB_10795709), for in-house IHC validation in human placental tissues. However, these antibodies did not produce sufficiently consistent and reproducible staining under the conditions tested (Supplementary Figure S2); therefore, the IHC validation was discontinued, and no staining data generated using either antibody are presented in the main manuscript. The quality assurance data were included in the Supplemental Materials (Supplementary Figure S2). The placental FADS1 IHC images were obtained from the publicly available Human Protein Atlas (version 25.0, access data is 03/01/2026)^84^ and are presented as external reference data.

### Statistics

#### Statistical analysis of transcriptomic data

Hardy–Weinberg equilibrium (HWE) for *FADS1* SNPs was evaluated using Chi-Square Goodness-of-Fit Test by comparing observed and expected genotype frequencies. Associations between *FADS1* genotypes and placental vascular outcomes, including PFVM and its subtypes, were examined using logistic regression models, from which odds ratios (ORs) and 95% confidence intervals (CIs) were derived. Both unadjusted and multivariable models were constructed, with adjustment for maternal age, BMI, race, and GA as appropriate. For transcriptomic analyses, gene expression values were log_2_-transformed prior to analysis, and associations between PFVM (or specific PFVM subtypes, such as SK) and placental gene expression were assessed using analysis of covariance (ANCOVA) or linear regression–based models with PFVM status as the primary factor. All statistical tests were two-sided, and exact P values are reported.

The genotypes of two SNPs of the *FADS1* locus (rs174538 and rs174556) were coded into 0, 1, and 2 based on the number of major alleles, corresponding to their association with *FADS1* gene expression levels low, medium, and high. The relationship between the genotype of each SNP and placental vessel pathologies was analysed using binary logistics and multiple logistic regression, controlling covariates such as maternal BMI, age, race, and GA by R studio. A p-value of less than 0.05 was considered statistically significant. A regression coefficient of less than 0 indicated that increased *FADS1* expression was associated with a decreased risk for PFVM pathology, while a coefficient greater than 0 suggested that reduced *FADS1* activity was correlated with an increased risk of pathology.

Similarly, correlations between PFVM, SK, or SNP genotypes (rs174538 and rs174556) and transcriptomic data were also analysed using multiple linear regression, adjusting for BMI, age, race, and GA, based on a p-value threshold of 0.05. The human placental and HUVEC transcript abundance from RNA-seq reads was quantified and used to identify differentially expressed genes (DEGs) with the housekeeping gene-normalised count per million (CPM). Genes whose expression are correlated with SNP genotypes, PFVM, or PFVM-SK (a liberal p-value cut-off of 0.05 was used to select the genes, given the limited number of significant genes) were further analysed for pathway enrichment using the ShinyGO 0.82^85^ pathway analysis tool (South Dakota State University) to identify associated biological pathways and networks. The statistical significance of enrichment was based on a cut-off of false discovery rate (FDR) of 0.05 for enriched pathways. The pathway network was analysed and performed using the ShinyGO 0.82 pathway network analysis tool (Edge cutoff 0.3–0.6). The significantly associated genes were also analysed for cell type specificity as well as enrichment for relevant metabolomic profiles using Enrichr (version 3.4)^86^, ^87^, ^88^ by comparing the profiles with the App for Reference-based Single Cell Analysis (AZIMUTH, version 2023) of the HuBMAP (version 2.0)^89^ and the Human Metabolome Database (HMDB),^90^^,^^91^ respectively. Adjusted p < 0.05 was used as a cut-off for significance. For mRNA transcriptome array test, differentially expressed genes (DEGs) were normalised by calculating z-scores for each gene to generate heatmaps.

Functional enrichment analyses were performed using Enrichr. Differentially expressed genes were analysed for metabolite set and cell-type enrichment, and enrichment results were ranked using the Enrichr combined score, which integrates the enrichment p-value and z-score to reflect both statistical significance and enrichment magnitude. Logistic regression analyses were used to estimate ORs and corresponding 95% CIs, whereas ORs and CIs are not applicable to Enrichr-based enrichment analyses. For combined score in Enrichr, they refer to the composite enrichment score generated by Enrichr for metabolite set or cell type enrichment analysis, rather than a clinical or pathological scoring system. This score integrates both the statistical significance (p-value) and the magnitude of enrichment (z-score) to rank enriched metabolites or cell type associated with PFVM (or SK, *FADS1* SNP)-related DEGs. Higher combined scores indicate stronger and more robust enrichment. Gene and protein expression of *FADS1* in human placenta was evaluated based on four publicly available single cell RNA sequencing databases^84^^,^^92^, ^93^, ^94^ and immunohistochemistry staining images available in the Human Protein Atlas (Human Protein Atlas proteinatlas.org).^84^

#### Statistical analysis of HTR-8s and HUVEC cell data

All cell samples were collected from at least 3–4 independent experiments. DAPI, EdU, and PI-positive cells were counted using the cell counter plugin in ImageJ, and the percentage of positive cells was calculated as (number of positive cells) × 100%/(number of DAPI-positive cells). For statistical analysis, data from in vitro studies were analysed using GraphPad Prism software (version 10.1) (Boston, MA, USA), with Welch's t tests or one-way ANOVA followed with *post-hoc* Tukey multiply comparisons. p-values less than 0.05 were considered statistically significant. Diagrams were generated using BioRender (Liu, Z. (2026) https://BioRender.com/xj84g4p; Science Suite Inc., Toronto, ON, Canada).

#### Sample size justification

The sample size for this translational study was determined by the availability of placental specimens with matched maternal genotype, placental histopathology, and transcriptomic data from an established pregnancy cohort. Maternal genotyping was performed in 646 pregnant women with available placental tissue, providing adequate representation of common and minor alleles for the two *FADS1*-linked SNPs rs174538 and rs174556. Placental histopathological evaluation was available for 168 samples, among which 53% were diagnosed with PFVM, yielding sufficient case–control balance for genetic association analyses with PFVM and its major histologic subtypes. Transcriptomic profiling was available for 102 placentas, and a subset of 73 placentas contained both RNA-sequencing and histological data, enabling integrative analyses linking maternal genotype, placental pathology, and gene expression. Given the observed genotype distribution and PFVM prevalence, *post-hoc* simulation-based power analyses indicated that the available complete-case sample size provided limited power to detect small genotype effects (25.9%–30.5% power for OR = 1.5), moderate power to detect odds ratios around 2.0 (58.0%–63.4% power), and higher power to detect larger effects (74.9%–81.8% power for OR = 2.5) under an additive genetic model adjusting for maternal age, BMI, race, and GA. The consistency of effect direction across both SNPs, persistence of associations after covariate adjustment, and concordance with known functional effects of *FADS1* variants on PUFA metabolism further support the adequacy of the sample size for the primary study objectives. For in vitro cell-based experiments, endothelial cells and trophoblast cell lines were used to assess cell viability, proliferation, tube formation, migration, and invasion, as well as *FADS1* knockdown–dependent effects on angiogenic tube formation using monoculture and endothelial–trophoblast co-culture systems, together with growth factor release assays. All in vitro analyses were conducted using at least three to four independent biological experiments per condition, with multiple technical replicates within each experiment, ensuring reproducibility and robustness of the observed effects.

#### Missing data

Of the 646 pregnant women, two did not have a valid rs174538 genotype, resulting in 644 available rs174538 genotypes; rs174556 genotypes were available for all 646 women. Missing data were confined to covariates included for adjustment (maternal BMI, age, race, and GA), while *FADS1* genotypes and PFVM outcomes were complete for all participants included in regression analyses. Because missingness did not affect the exposure or outcome variables and sensitivity analyses showed minimal impact of covariate adjustment on effect estimates, complete-case analysis was used for the primary regression models. This approach minimised model complexity while preserving interpretability of the exposure–outcome association. For missing data, while 646 pregnant women were genotyped, only 168 participants had information on placental pathology, 102 with transcriptomic data, and 73 with both pathological and transcriptomic data (Supplementary Figure S3B). Therefore, regression analyses were focused on the 197 samples with both genotypic data and at least one of pathological or transcriptomic data. Among these 197 participants, the proportion of missing data across predictors was 0% for maternal age (0/197), 8.1% for race (16/197), 9.6% for BMI (19/197), and 12.7% for gestational age (25/197), corresponding to an overall range of 0–12.7% missing across predictors. In total, 45 of 197 participants (22.8%) had at least one missing predictor.

### Role of funders

The funding sources had no role in the study design; data collection, analysis, or interpretation; writing of the manuscript; or the decision to submit the manuscript for publication.

## Results

### Low-function SNP alleles are associated with increased risk of PFVM in human placenta

Overall, histopathology of placental samples from 168 pregnant women were reviewed by an experienced pathologist in our team (C.S.). The transcriptome of 102 placentas was profiled using mRNA transcriptome array. Among these samples, 73 placentas had both transcriptomic profiles and histological data, enabling us to evaluate the relationships between PUFA desaturation, PFVM, and transcriptomic changes. Among 168 samples reviewed for histology, 89/168 (53%) were diagnosed with FVM. Among which, placentas were diagnosed with either a single or mixed FVM subtypes, with 67/168 (40%) were diagnosed with AVV, 30/168 (18%) with SK, 15/168 (9%) with HEV, 25/168 (15%) with FTHR, and 15/168 (9%) with CPTHR (Table 1 and Supplementary Figure S3A). Analyses between PFVM and demographic data suggest that PFVM is marginally associated with older age and White race of the pregnant women, but not with gestational age, preterm rate, or mother's BMI (Table 1).

**Table 1:** Demographic information and associations with PFVM.

Category	Control	PFVM	p-value
Total patient number (%)	79 (47%)	89 (53%)	–
PFVM subtypes			
AVV (%)		67 (40%)	–
SK (%)		30 (18%)	
HEV (%)		15 (9%)	
FTHR (%)		25 (15%)	
CPTHR (%)		15 (9%)	
Mother age median (range)	28 (18–45)	29 (18–51)	0.0444^a^
Mother race/ethnicity			
White (%)	43 (59%)	60 (75%)	0.0394^b^^,^^c^
African American (%)	29 (40%)	13 (16%)	
Other (%)	1 (1%)	7 (9%)	
GA median (range)	39 (28–41)	39 (33–42)	0.316^a^
Preterm rate (GA <37 wks)	15% (11/75)	10% (8/83)	0.463^b^
Mother BMI median (range)	26.6 (16.6–75.1)	24.8 (17.6–53.0)	0.143^a^

AVV = avascular villi; SK = stromal karyorrhexis; HEV = hemorrhagic endovasculitis; FTHR = foetal stem mural thrombosis; CPTHR = chorionic plate vessel mural thrombosis.

The number of participants with available data varies by variable. Percentages were calculated using participants with nonmissing data for each variable.

a Statistical analysis was based on Mann–Whitney U test.

b Statistical analysis was based on Fisher’s exact test.

c Statistics for race is based on White vs non-White. Nominal p values of the association analyses were shown.

We focused primarily on the role of PUFA biosynthesis, the crucial metabolic process that produces key long-chain n-3 (EPA and DHA) and n-6 PUFAs (ARA) (Fig. 1)^34^^,^^56^^,^^57^ in placental health during pregnancy. We first hypothesised that genetically impaired LC-PUFA production is a risk factor for PFVM. To test this hypothesis, we genotyped two representative SNPs rs174538 in 644 and rs174556 in 646 pregnant women whose placentas were collected after birth (Table 1 and Supplementary Figure S3B). The GTEx database analysis and our results showed that the mRNA expression of *FADS1* was associated with *FADS1* SNPs both in human liver and placenta (Supplementary Figure S1A–D). As noted above, both SNPs have been well-documented for their high correlation with both *FADS1* gene expression and circulation levels of ARA, EPA and DHA.

Our results showed that at the rs174538 locus, 29/644 (4.5%) pregnant women carry the minor allele homozygous genotype (A/A), 191/644 (29.7%) are heterozygous (A/G), and 424/644 (65.8%) have the homozygous genotype of the common allele (G/G). Statistical analysis did not show a significant deviation from the Hardy–Weinberg equilibrium (HWE with Chi-Square Goodness-of-Fit Test, p = 0.21). Similarly, at the rs174556 locus, 31/646 (4.8%) pregnant women carry the minor allele homozygous genotype (T/T), 191/646 (29.6%) are heterozygous (T/C), and 424/646 (65.6%) have the homozygous genotype of the common allele (C/C) (HWE with Chi-Square Goodness-of-Fit Test p = 0.12).

To analyse the association between *FADS1* SNPs and PFVM, a directed acyclic graph (DAG) was constructed to identify potential confounders, including maternal BMI, age, race, and GA (Supplementary Figure S4). Although the analysis suggested that these variables were not true confounders in the relationship between *FADS1* SNPs and PFVM (Supplementary Table S3–S6), they were still included in the model to improve the accuracy and robustness of the analysis. Then, we performed univariate followed by binary logistic and multiple logistic regression analysis, controlling maternal BMI, age, race, and GA, to examine the association between SNP genotypes and PFVM (Fig. 2A). The exploratory associations between SNPs and various PFVM histology sub-features including AVV, SK, HEV, FTHR, and CPTHR were also conducted. Our analysis indicated that rs174538 was significantly associated with PFVM (p = 0.039 and adjust p = 0.0011 by controlling covariables, coefficient = −1.59, stand error = 0.77, z value = −2.06, after adjusting for all covariables; Fig. 2B). Similar analysis further demonstrated that rs174538 was also significantly associated with SK (p = 0.033 and adjust p = 0.020 by controlling covariables, coefficient = −1.21, standard error = 0.57, z value = −2.13), with the carries of the minor allele showing a higher pathological score (Fig. 2C). This association clearly demonstrated an additive effect of the minor allele. Similar trends and differences were also observed with rs174556, though the associations were slightly weaker (Fig. 2D and E). Given the limited sample size of the minor allele homozygous group (less than 10), we further analysed the data by combining the minor allele homozygote and heterozygote carriers to compare their risk for PFVM with the major allele homozygote carriers. Similar statistical trends were also observed (rs174538: p = 0.00094, adjust p = 0.0038 by controlling covariables, coefficient = −0.79, stand error = 0.24, z value = −3.31; rs174556: p = 0.035, adjust p = 0.088 by controlling covariables, coefficient = −0.49, standard error = 0.23, z value = −2.11; Supplementary Figure S5). As mentioned earlier, since the minor allele of these SNPs are associated with decreased FADS1 function as well as decreased levels of LC-PUFAs among humans, our data suggest that reduced maternal PUFA desaturation activity or lower circulatory LC-PUFA levels are associated with increased risk for PFVM, particularly SK, in pregnant women.

**Fig. 2:**
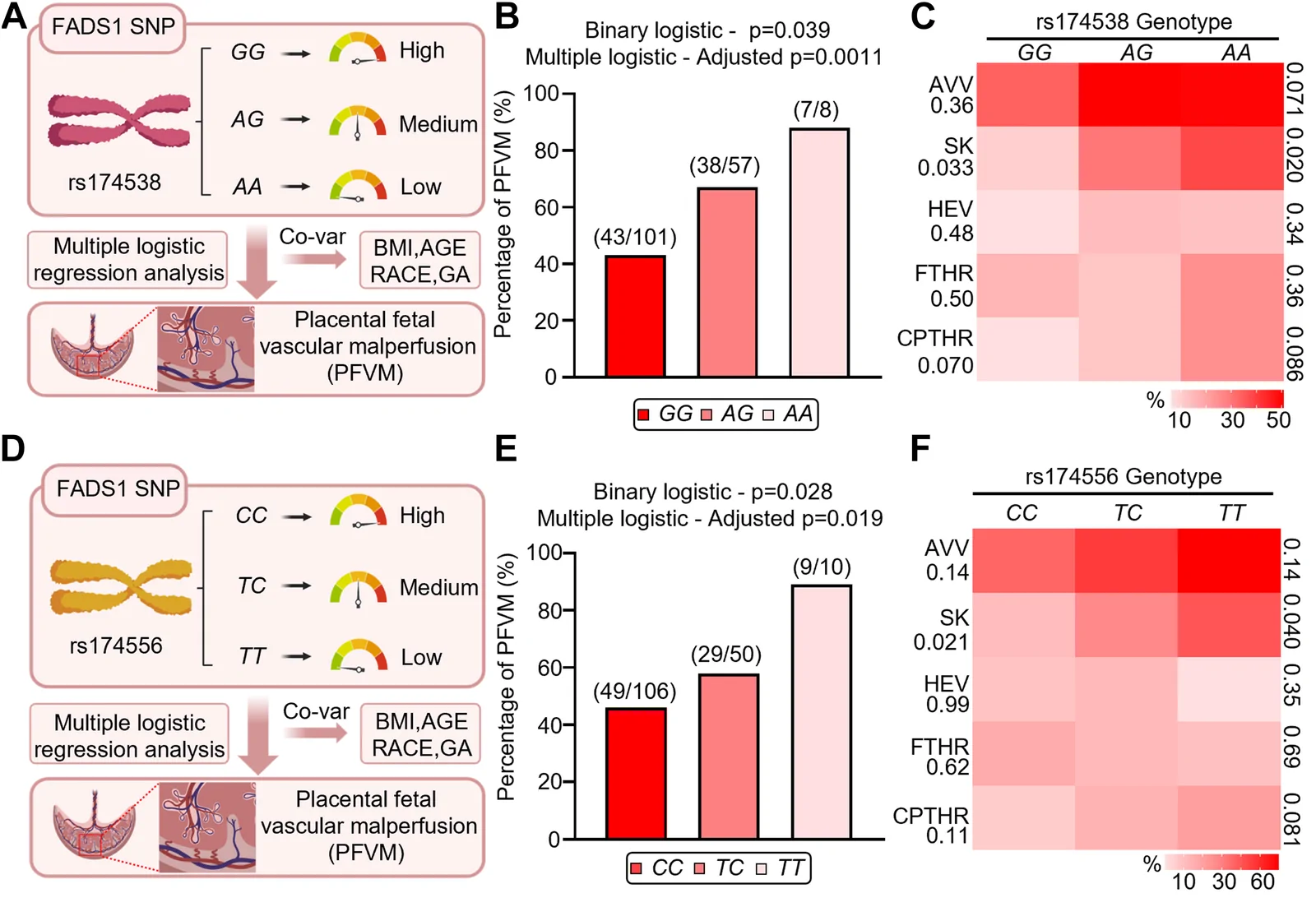
**Low-functional alleles of maternal FADS1 are associated with placental foetal vascular malperfusion (PFVM).** (A) Schematic diagram depicted the analysis of the relationship between *FADS1* low-function alleles (A for rs174538, D for rs174556) and PFVM using binary logistic regression and multiple logistic regression analysis. Covariables of multiple logistic regression analysis, including maternal age, race, body mass index (BMI), and gestational age (GA) were excluded to ensure accuracy. (B) Column bar graph showed the percentage of PFVM across different levels of FADS1 genetic expression for rs174538 (E for rs174556) (n = 166). Statistical analysis was conducted using binary logistic regression (nominal p value on the left) and multiple logistic regression (adjusted p value on the right). Adjusted p-values reflect statistical inference after accounting for potential confounding factors. (C) The PFVM, including Avascular Villi (AVV), Stromal Karyorrhexis (SK), Hemorrhagic Endovasculitis (HEV), Foetal Stem Mural Thrombosis (FTHR), and Chorionic Plate Vessel Mural Thrombosis (CPTHR), were scored by their immunohistology information. The heatmap illustrates the relationship between pathology scores and FADS1 genetic expression level of rs174538 (F for rs174556) (n = 166). Statistical analysis was conducted using binary logistic regression (nominal p value on the left) and multiple logistic regression (adjusted p value on the right). Adjusted p-values reflect statistical inference after accounting for potential confounding factors.

### PFVM-associated transcriptomic alterations confirmed canonical pathways and highlighted the linkage to PUFA metabolism and key cell types

To explore the genes or pathways that might be associated with PFVM, transcriptomic profile was compared between placentas with and without PFVM (Fig. 3A). Data analysis revealed 54 and 34 genes significantly down- and upregulated in placentas with PFVM, respectively (Fig. 3B). The DEGs include well-established markers for a few key pathways canonically deemed to be associated with PFVM (Fig. 3C). More specifically, key growth factors, such as *VEGFA*, *FGF2*, *TDGF1*, etc, were significantly reduced in the PFVM placenta, indicating a potentially impaired endothelial cell proliferation and migration. The developmental angiogenic regulators, including *SHH*, *TBX3*, *BRCA1*, and *HDAC7*, were also significantly reduced, suggesting impaired blood vessel development. Furthermore, several anti-apoptotic and cell survival genes, including *FLT3*, *KITLG*, ASNS, *AKAP*, *ADIPOQ*, and *SNAI1*, exhibited significant downregulation. Meanwhile, there were also six apoptotic genes showing significant increase, including *CYLD, CTSC, CREB3L1, CX3CR1, C1QA*, and *SYK.* All these DEGs suggested enhanced cell death stimulation and exacerbated PFVM-related placental vessel damage during pregnancy. Lastly, the macrophage/phagocyte markers and activity genes (*CD68*, *CD163*, *LYZ*, *CYBB*, *PLA2G2A*, and *A2M)*, were significantly increased, while the inflammatory signalling gene (*RIPK2*), leucocyte recruitment gene (*CCL20*, *CXCL13*, *CXCR5*, and *SELE*), and immune tolerance genes (*IL10*, *IDO2*, and *MST1*) were significantly decreased. These alteration of the immune response associated genes were also associated with and relevant to PFVM induced cell death and anti-angiogenesis in the placenta. Gene-set enrichment analysis also confirmed that the DEGs were enriched to pathways related to immune response, blood vessel development, cell death, etc (Fig. 3C–E). Taken together, these results suggested that PFVM is associated with impaired blood vessel development and cellular homoeostasis.

**Fig. 3:**
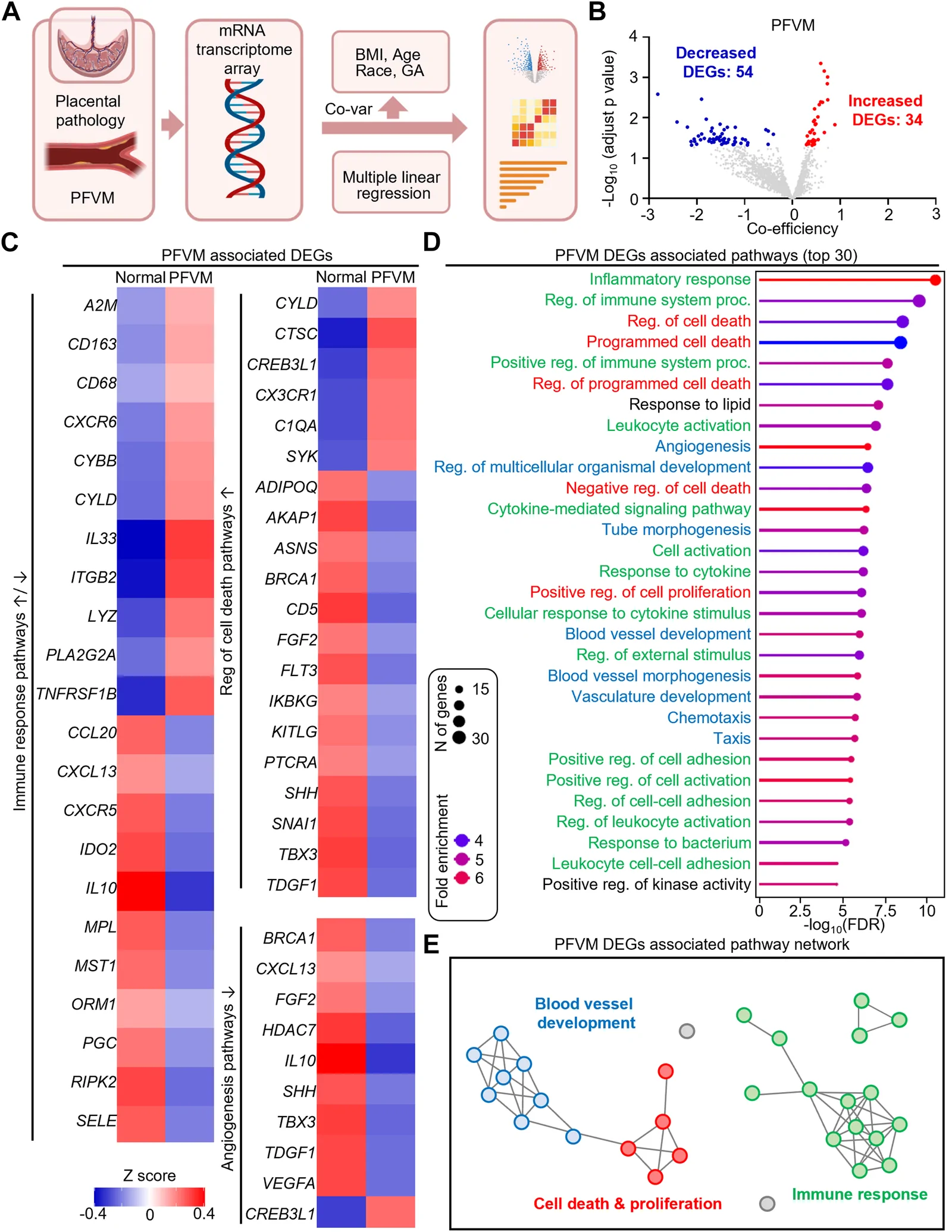
**Altered transcriptomic correlated with PFVM.** (A) Schematic diagram depicted the analysis of the relationship between PFVM and placental transcriptomic changes using multiple linear regression analysis. Covariables such as maternal age, race, BMI, and GA were excluded to ensure accuracy. (B) Volcano graph displayed 34 DEGs that showed a significant increase in expression as PFVM in placental samples (n = 73), while 54 DEGs showed a significant decrease. (C) Heatmap showed selected DEGs and the enriched pathway associated with PFVM. (D) Graph illustrated the top 30 pathways activated by differentially expressed genes (DEGs) associated with PFVM. A False Discovery Rate (FDR) of 0.05 was used as the threshold for identifying significant pathways. (E) Pathway network graph illustrated the clusters of associated pathways, with the main cluster pathway focused on in this study indicated in blood vessel development (blue in D), cell death (red in D), and immune response (green in D). p values for enrichment analyses were adjusted using the FDR method, and FDR-adjusted p value < 0.05 were considered statistically significant.

*FADS1* is a key enzyme controlling metabolism of LC-PUFAs, especially ARA and EPA. Studies have also demonstrated that *FADS1* polymorphisms further determine the lipids derivatives of these LC-PUFAs, especially those of lipids metabolites of ARA.^95^ Therefore, it would be interesting to examine the metabolomic profile in the placenta samples. However, our formalin-fixed and paraffin-embedded (FFPE) samples do not allow us to perform metabolomic or lipidomic analysis. We then performed a bioinformatic gene-metabolome enrichment analysis to investigate the potential metabolites that are most likely enriched for the differentially expressed genes (DEGs) signatures that are associated with PFVM. Interestingly, ARA and many ARA derivatives are top enriched metabolites that are associated with the PFVM-associated DEGs (Supplementary Table S7), suggesting alterations in LC-PUFA metabolism underlying the transcriptomic changes associated with PFVM.

Given the complexity of histological organisation of placenta, we set out to understand the dominant cell types that are involved in foetal development of PFVM pathogenesis. We then performed a cell type specificity enrichment analysis based on the PFVM-associated DEGs. Our analysis highlighted endothelial cells as top enriched cell type (Supplementary Table S8).

Given that SK is the subtype of histological changes most significantly associated with *FADS1* genotypes, we further explored the transcriptomic profile changes associated with SK (Fig. 4A). We found that 17 and 52 genes significantly down- and upregulated in placentas with SK, respectively (Fig. 4B and C). Enriched top pathways based on the SK-associated DEGs include cell death, cell cycle, cellular response to lipid etc (Fig. 4D and E). Notably, several typical genes involved in cell survival or cell proliferation signalling showed a significant decrease in the SK group, including *BIRC3* and TNFRSF12A, which activate anti-apoptotic signalling pathway; *FLT4* and *PIK3CG*, which promote endothelial survival, proliferation, and migration; *MLH1*, which support long-term cell survival; as well as *KDM1A*, which regulate survival and proliferation via epigenetic pathway. Conversely, several genes involved in cell death and cell cycle arrest signalling show a significant increase, including *CASP6*, which induce apoptosis execution; *BCL2L11* and *MAPK8*, which promote intrinsic apoptosis; *ATG5*, which regulates autophagy; LTF and MAGEA4, which modulate apoptosis through inflammatory pathways; *GAS1* and *AXIN2*, which involve in growth arrest; as well as *MAD2L1*, which are associated with cell-cycle checkpoint (Fig. 4C). Furthermore, cell type specificity analysis also identified that various endothelial cells are top enriched cell type associated with the SK-associated DEGs in foetal development (Supplementary Table S9).

**Fig. 4:**
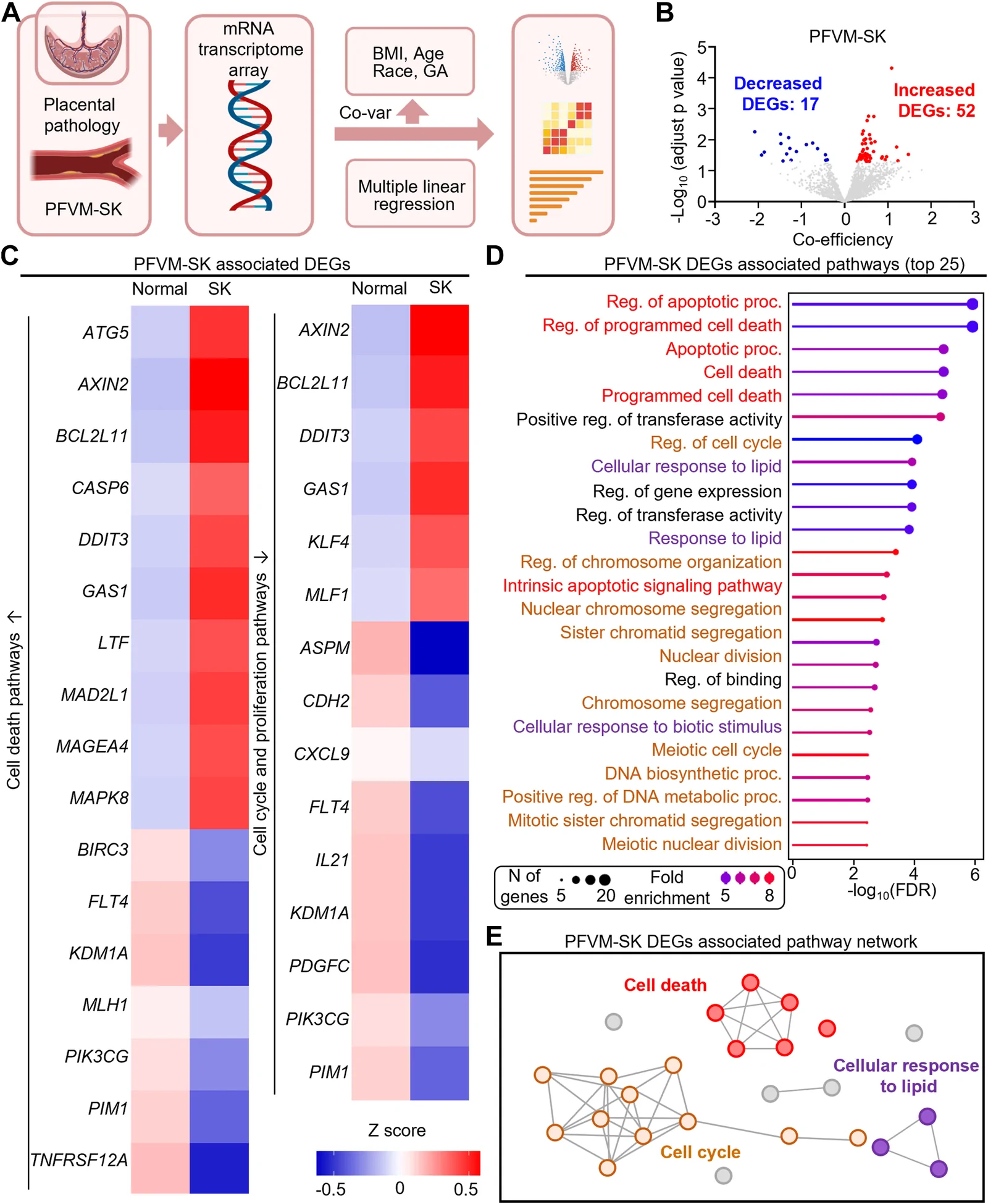
**Altered transcriptomic correlated with stromal karyorrhexis (SK) in the placenta.** (A) Schematic diagram depicted the analysis of the relationship between PFVM-SK and placental transcriptomic changes using multiple linear regression analysis. Covariables such as maternal age, race, BMI, and GA were excluded to ensure accuracy. (B) Volcano graph displayed 52 DEGs that showed a significant increase in expression as PFVM-SK in placental samples (n = 73), while 17 DEGs showed a significant decrease. (C) Heatmap showed selected DEGs were associated with PFVM-SK, which were relative with cell death, cell cycle, and proliferation pathways. (D) Graph illustrated the top 25 pathways activated by DEGs associated with PFVM-SK. An FDR of 0.05 was used as the threshold for identifying significant pathways. (E) Pathway network graph illustrated the clusters of associated pathways, with the main cluster pathway focused on in this study indicated in cell death (red in D), cell cycle (orangeoran in D), and cellular response to lipid (purple in D). p values for enrichment analyses were adjusted using the FDR method, and FDR-adjusted p value < 0.05 were considered statistically significant.

To further verify the involvement of maternal and foetal cell types in the pathogenesis of PFVM, we analysed the multiple publicly available databases to examine the expression pattern of *FADS1* in the maternal foetal interface. Single-cell RNA-sequencing and IHC staining results consistently showed high expression of *FADS1* gene and protein in endothelial cells, trophoblasts, as well as maternal decidua cells (Supplementary Figure S6).^84^^,^^92^, ^93^, ^94^^,^^96^ Taken together, these data well confirmed the involvement of multiple molecular pathways e.g. endothelial cell cycle, apoptosis & angiogenesis, cellular response to stimulate, inflammatory and immune modulation, and highlighted the key role of placental cells at the maternal–foetal interface in the development of PFVM.

### *FADS1* genotype-associated transcriptomic profile consistent with that of PFVM and further highlighted the involvement of endothelial cells

To investigate the potential mechanism underlying the association between *FADS1* SNPs and PFVM, we examined the correlation between rs174538 or rs174556 genotypes and the transcriptomic data among 102 placenta tissue samples by adjusting covariables. We found that 114 and 5 genes demonstrated a significantly positive and negative correlation with the minor allele of rs174538, respectively, while 136 and 4 genes demonstrated a significantly positive and negative correlation with the minor allele of rs174556 (Fig. 5A–C). Among these DEGs for the minor allele of rs174538 and rs174556, there were also 48 DEGs which are shared between these two SNPs (Fig. 5D). These 48 shared DEGs are significantly enriched in pathways including cell proliferation, cell death, regulation of metabolic, blood vessel remodelling, etc (Fig. 5E and F). More specifically, several key apoptosis-associated genes, including *BCL2L11, TNF, CYCS, DPYSL4,* and *DAPL1,* etc. showed a significant association with *FADS1* low-function allele across the two SNPs. Also, there is a significant association between expression of *NLRP3*, the master regulator of the inflammatory response, and the *FADS1* low-function alleles, further confirming that immune signalling is altered under different levels of *FADS1* expression in the placenta. Notably, there were 6 DEGs which were associated with both *FADS1* SNPs (rs174538 and rs174556) and SK, including *BCL2L11, DUSP4, IL21, MFNG, MRPL13,* and *PLAT*. These genes are hallmarks for cell death, immune response, angiogenesis and vascular remodelling, and cellular metabolism. Furthermore, to understand the major tissue/cell types underlying these associated genes, we performed cell specificity enrichment analysis for the DEGs that are associated with different SNPs. Endothelial cells are again dominantly enriched for these genes (Supplementary Table S10).

**Fig. 5:**
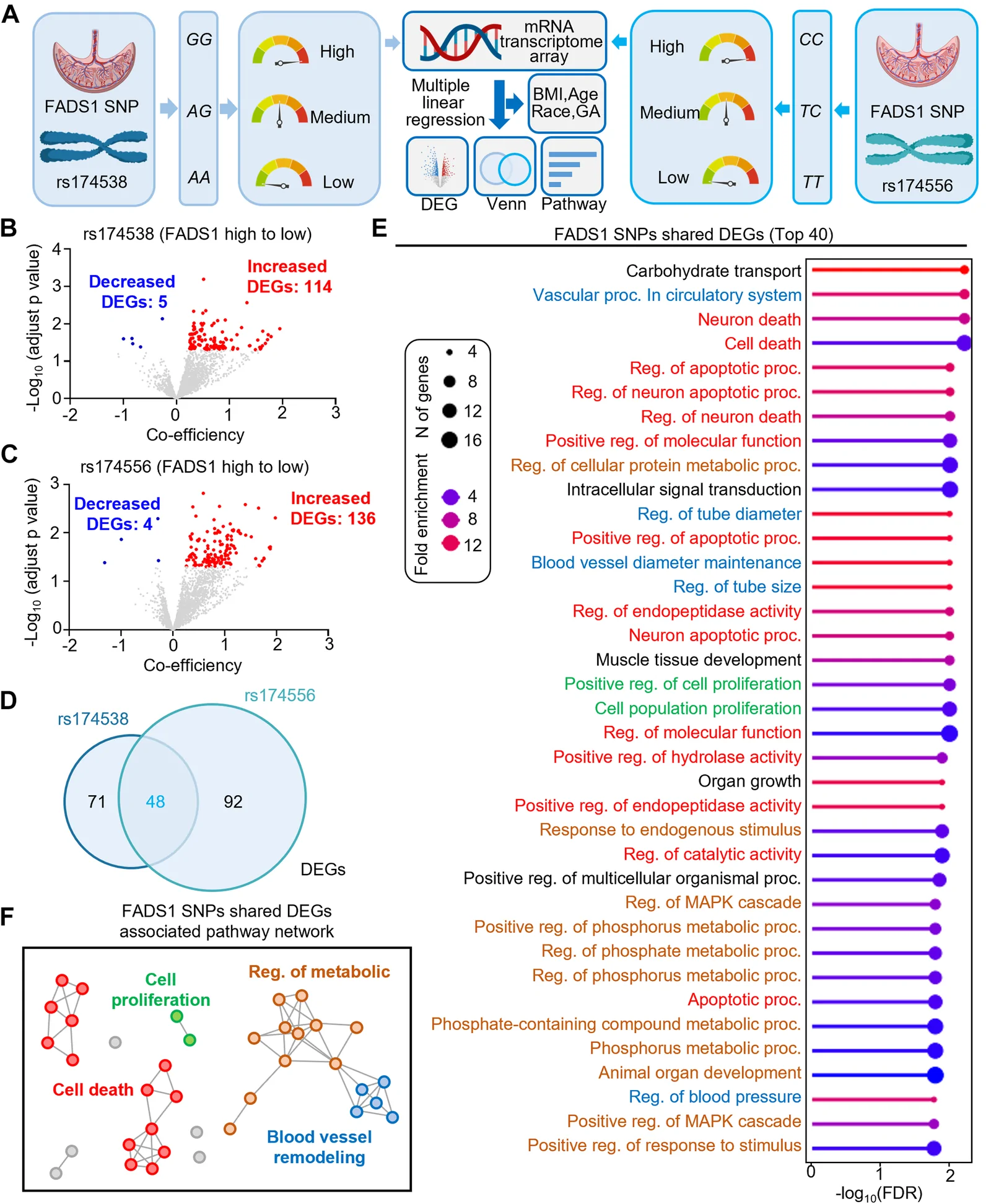
**Altered transcriptomic correlated with low-function alleles of FADS1 in the placenta.** (A) Schematic diagram depicted the analysis of the relationship between *FADS1* genotypes and placental transcriptomic changes using multiple linear regression analysis, focussing on *FADS1* SNP rs174538 and rs174556. Covariables such as maternal age, race, BMI, and GA were excluded to ensure accuracy. (B) Volcano graph displayed 114 upregulated and 5 downregulated DEGs that showed a significant association with FADS1 low-function alleles of rs174538 (n = 102). (C) Volcano graph displayed 136 upregulated and 4 downregulated DEGs that showed a significant association with the low function allele of rs174556 in placental samples (n = 102). (D) Venn plot showed there were 48 DEGs shared in the *FADS1* SNP rs174538 and rs174556. (E) Graph illustrated the top 40 pathways altered by shared DEGs associated with reduced FADS1 expression. A FDR of 0.05 was used as the threshold for identifying significant pathways. (F) Pathway network graph illustrated the clusters of associated pathways, with the main cluster pathway focused on in this study indicated in cell proliferation (green in E), cell death (red in E), regulation of metabolic (orange in E), and blood vessel remodelling (blue in E). P values for enrichment analyses were adjusted using the FDR method, and FDR-adjusted p value < 0.05 were considered statistically significant.

Taken together, low-function alleles of *FADS1* polymorphisms are associated with transcriptomic patterns related to signalling pathways involving cell death, cell cycle, vessel development and remodelling, as well as inflammation and immune modulation, which is highly consistent with the transcriptomic profiles associated with PFVM. Meanwhile, these gene expression patterns strongly suggest the involvement of endothelial cells, matching the pathological features of PFVM.

### LC-PUFA exposure and alterations in *FADS1* function in maternal–foetal interface altered cell function and cell–cell interaction in vitro

We further aim to understand the molecular mechanism underlying the various associations observed above, especially the mechanism about why maternal genotype influences the risks for foetal vascular malperfusion. Previous studies have well established that foetal circulating/cellular LC-PUFA levels are fundamentally determined by the LC-PUFA profiles of the maternal system, while maternal systemic LC-PUFA levels are significantly associated with *FADS1* genotype.^69^, ^70^, ^71^, ^72^, ^73^, ^74^ Also, studies have demonstrated that foetal *FADS1* activity indexed by the ARA/DGLA ratio is predominantly associated with maternal *FADS1* genotypes.^61^^,^^62^ We have further demonstrated above that placental *FADS1* mRNA expression in our samples is also associated with maternal *FADS1* genotype (Supplementary Figure S1C and D), while placental *FADS1* expression is also negatively associated with PFVM (Supplementary Figure S1E). Therefore, it is reasonable that cellular levels of *FADS1* and its LC-PUFA products at the maternal–foetal interface are primarily controlled by the maternal *FADS1* genotype. Also, foetal cells exposed to different levels of maternal circulating LC-PUFAs may exert different functionality that is related to the risk for PFVM. As noted above, the transcriptomic profiles associated with PFVM and *FADS1* genotype strongly suggested the involvement of endothelial cells. Meanwhile, trophoblasts are known to play critical role at the maternal–foetal interface, especially in supporting foetal endothelial cell function and blood vessel development.^97^^,^^98^ Indeed, single-cell RNA sequencing and IHC results from publicly available databases have demonstrated high *FADS1* mRNA or protein expression in both endothelial and trophoblast cells (Supplementary Figure S6). We therefore explored the influence of exogenous LC-PUFAs and endogenously altered *FADS1* function in both endothelial cells (HUVEC) and trophoblast cell line.

Since pregnant women carrying the major *FADS1* allele have higher systemic level of LC-PUFAs, including ARA and DHA, we first hypothesise that such a LC-PUFA-rich microenvironment in the placenta supports vascular development and remodelling by regulating trophoblast and endothelial cell function. To test this hypothesis, trophoblasts were treated with gradient concentrations of LC-PUFAs (ARA and DHA in 1:1 ratio), followed by evaluation of cell invasion, growth factor secretion, apoptosis, and proliferation. Our results showed that treatment with such a balanced n-3 and n-6 LC-PUFA cocktail significantly increased trophoblast invasion (Fig. 6A–C) and the secretion of angiogenic factors, including VEGF-A and PlGF (Fig. 6E), which are key stimulators for endothelial tube formation function. However, LC-PUFA treatment did not affect trophoblast proliferation (Fig. 6F and G) or cell viability (Fig. 6I and J). Similarly, in HUVECs, LC-PUFA treatment significantly enhanced cell migration (Fig. 7A–C) and tube formation (Fig. 7E and F). However, it did not affect endothelial cell proliferation (Fig. 7H and I) or viability (Fig. 7K–L). Together, these findings indicate that an LC-PUFA-rich microenvironment indeed modulates trophoblast and endothelial cell function and possibly promotes placental vascular development.

**Fig. 6:**
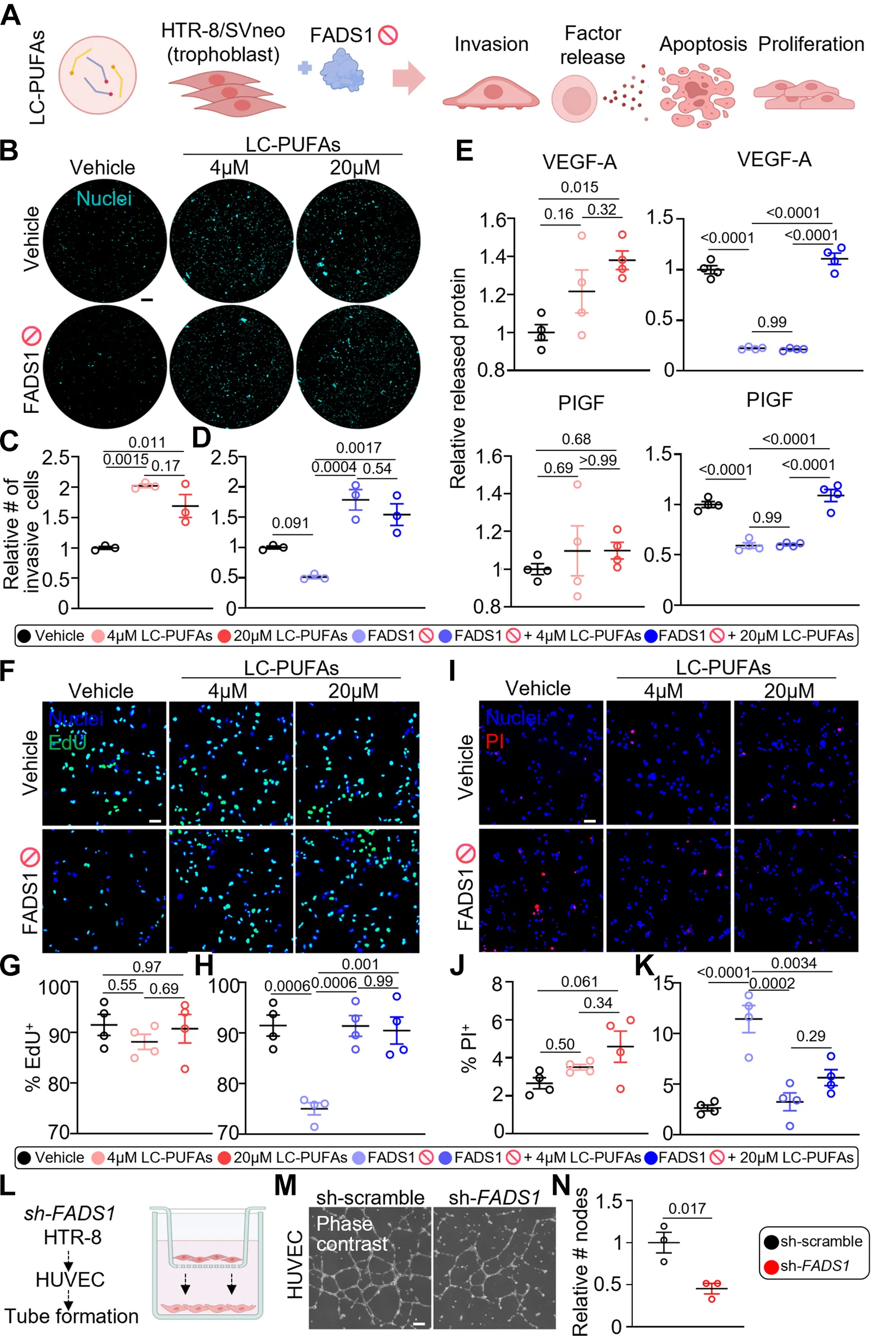
**LC-PUFAs treatment and/or inhibition of FADS1 altered the trophoblasts' function and their interaction with endothelial cells.** (A) Schematic diagram illustrated HTR-8/SVneo (trophoblasts) were treated with LC-PUFAs (ARA and DHA) with or without FADS1 inhibitor D5D-IN-326. (B) HTR-8 cells were treated with LC-PUFAs (ARA-BSA and DHA-BSA, 4 or 20 μM, BSA as control) with or without D5D-IN-326 (2 μM, DMSO as control) and cultured in the upper level of Matrigel coated transwell plate for 24 h. The representative immunofluorescence images showed the overview of migrated HTR-8 cells. Scar bar is 1 mm. (C and D) The dot graph indicated the total number of migrated cells with different types of treatments at 24 h (mean ± standard error). (E) The dot graph indicated the release growth factors (VEGF-A and PIGF) in the HTR-8 cells medium with different treatment (mean ± standard error). (F) The representative immunofluorescence images showed the expression of EdU in HTR-8 with different types of treatments. Scar bar is 50 μm. (G and H) The dot graph indicated the percentage of EdU positive cells in the different types of treated HTR-8 cells at 24 h (mean ± standard error). (I) The representative immunofluorescence images showed the expression of the PI in HTR-8 with different types of treatments. Scar bar is 50 μm. (J and K) The dot graph indicated the percentage of PI positive cells in the different type of treated HTR-8 cells at 24 h (mean ± standard error). (L) Schematic diagram illustrated sh-*FADS1* or sh-scramble virus-treated HTR-8 cells were seeded on the upper insert transwell plate and co-cultured with HUVEC (bottom) for 24 h. The tube formation condition of the HUVECs were evaluated and compared. (M) The representative phase contract images showed the tube formation condition in sh-scramble and sh-*FADS1* co-cultured HUVECs. Scar bar is 50 μm. (N) The dot graph indicated the relative number of nodes in all groups (mean ± standard error). Statistical analysis was performed based on one-way ANOVA followed by post-hoc Tukey multiply comparisons (C, D, E, G, H, J, and K) and Welch's t tests (N) (n = 3 for C and D; n = 4 for E, G, H, J, K, and N). All Tukey multiply comparisons or Welch's tests-adjusted p values were labelled in the plots. All experiments were performed in 3–4 independent replicates.

**Fig. 7:**
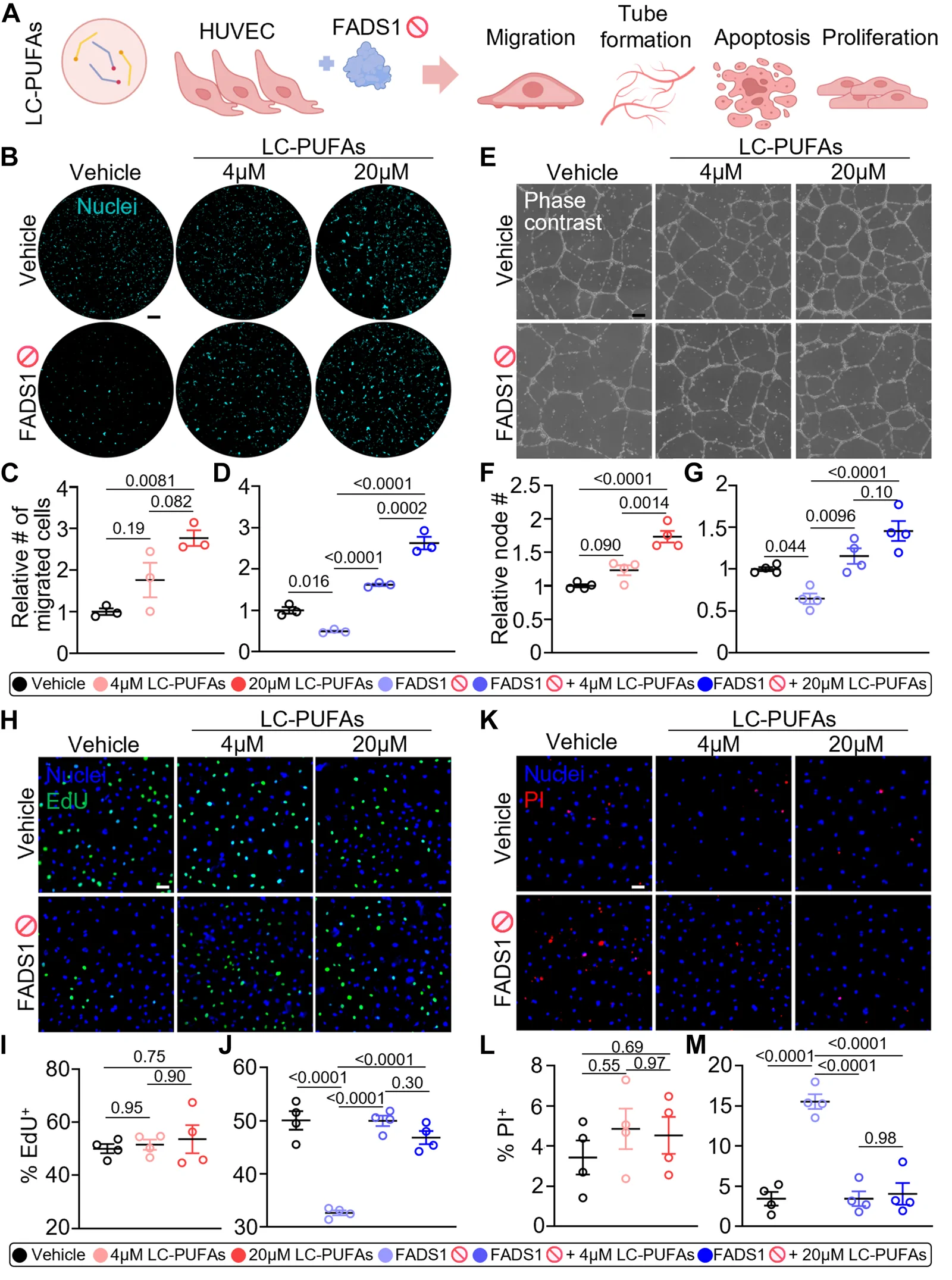
**LC-PUFAs treatment and/or inhibition of FADS1 altered the endothelial cell migration, tube formation, viability, and proliferation.** (A) Schematic diagram illustrated HUVECs (endothelial cells) were treated with LC-PUFAs (ARA and DHA) with or without D5D-IN-326, following with migration, tube formation, apoptosis, and proliferation evaluation. (B) HUVECs were treated with LC-PUFAs (ARA-BSA and DHA-BSA, 4 or 20 μM, BSA as control) with or without D5D-IN-326 (2 μM, DMSO as control) and cultured in the upper level of transwell plate for 24 h. The representative immunofluorescence images showed the overview of migrated HUVECs. Scar bar is 1 mm. (C and D) The dot graph indicated the total number of migrated cells with different types of treatments at 24 h (mean ± standard error). (E) The representative phase contract images showed the tube formation condition in all treated HUVECs. Scar bar is 50 μm. (F and G) The dot graph indicated the relative number of nodes in all groups (mean ± standard error). (H) The representative immunofluorescence images showed the expression of EdU in HUVECs with different types of treatments. Scar bar is 50 μm. (I and J) The dot graph indicated the percentage of EdU positive cells in the different types of treated HUVECs at 24 h (mean ± standard error). (K) The representative immunofluorescence images showed the expression of the PI in HUVECs with different types of treatments. Scar bar is 50 μm. (L and M) The dot graph indicated the percentage of PI positive cells in the different types of treated HUVECs at 24 h (mean ± standard error). Statistical analysis was performed based on one-way ANOVA followed by post-hoc Tukey multiply comparisons (n = 3 for C and D; n = 4 for F, G, I, J, L, and M). All p values adjusted with Tukey multiply comparisons were labelled in the plots. All experiments were performed in 3–4 independent replicates.

Interestingly, our data also showed that LC-PUFA treatment regulated *FADS1* expression in both trophoblasts and endothelial cells (Supplementary Figure S7), suggesting that the exogenous LC-PUFA profile could be critically involved in shaping the *FADS1* expression in foetal cells. Therefore, low exogenous LC-PUFA levels attributed to the low-function maternal *FADS1* genotype may lead to an insufficient induction of *FADS1* expression in foetal cells. This can be further exacerbated when foetal cells also carry the *FADS1* low-function genotype,^61^ while the foetus of a mother carrying low-function allele has a higher likelihood of carrying the low-function allele as compared to the mother who is a non-carrier. Unfortunately, our attempt to obtain the foetal genotype based on the available materials has failed. Nevertheless, the maternal *FADS1* genotype is significantly correlated with the placental level of *FADS1* mRNA among our samples (Supplementary Figure S1C and D), suggesting that the maternal genotype has a strong influence on *FADS1* expression in foetal cells as well. We then further hypothesised that reduced *FADS1* expression in trophoblasts and endothelial cells also contributes to impaired placental vascular development and remodelling via altering the endogenous level of LC-PUFAs. To mimic the reduced *FADS1* activity associated with the minor *FADS1* allele, cells were treated with a *FADS1* inhibitor followed by LC-PUFA supplementation to determine whether downstream lipid products could rescue the observed phenotypes. Our results showed that *FADS1* inhibition in trophoblasts reduced cell invasion (Fig. 6D), decreased VEGF-A and PlGF secretion (Fig. 6E), reduced proliferation (Fig. 6H), and increased apoptosis (Fig. 6K). Most of these effects were rescued by LC-PUFA treatment. Similarly, endothelial cells were also evaluated following *FADS1* inhibition. *FADS1* inhibition reduced endothelial cell migration (Fig. 7B and D), tube formation (Fig. 7E and G), and proliferation (Fig. 7H and J), while increasing apoptosis (Fig. 7K and M). Most of these effects were also rescued by LC-PUFA treatment. In addition, *FADS1* knockdown trophoblasts were generated and validated (Supplementary Figure S8A and B). *FADS1* knockdown also significantly reduced VEGF-A and PlGF expression (Supplementary Figure S8C) and directly impaired endothelial tube formation in the trophoblast–endothelial co-culture system (Fig. 6L–N). These findings indicate that endogenously reduced *FADS1* expression directly impairs maternal–foetal interface function by disrupting placental vascular development and remodelling, which can be rescued by exogenous supplementation of *FADS1* downstream products.

Collectively, our findings suggest that maternal *FADS1* genotype may contribute to PFVM risk through altering both exogenous and endogenous levels of LC-PUFAs of maternal–foetal interface cells, while exogenous supplementation of LC-PUFAs can rescue or improve the functions of these cells.

## Discussion

PFVM could be attributed to various factors such as stromal cell death, impaired blood vessel formation, and thrombosis induced by developmental abnormalities.^1^^,^^99^, ^100^, ^101^ Different stromal cell types, including trophoblast, endothelial cells, and macrophages, play critical roles in blood vessel formation.^97^^,^^102^, ^103^, ^104^, ^105^ Additionally, debris from deceased endothelial cells may trigger an immune response and promote thrombosis that further contributes to PFVM.^106^, ^107^, ^108^ This pathology significantly impairs blood flow, which is crucial for the proper delivery of nutrients and oxygen to the developing foetus.^2^^,^^12^ Consequently, PFVM is associated with several severe adverse pregnancy outcomes, such as foetal growth restriction, preterm birth, and even stillbirth, highlighting the long-term clinical impacts of this condition.^1^^,^^109^, ^110^, ^111^ However, the molecular mechanism underlying PFVM remains elusive.

In this study, we hypothesise that genetic variations in PUFA desaturation process influence placental health and function during pregnancy. As noted above, PUFAs, especially n-3 and n-6 LC-PUFAs e.g. ARA, EPA and DHA as well as their metabolic derivatives play critical roles in metabolic, immunological, and inflammatory processes and responses.^112^, ^113^, ^114^, ^115^ They are also pivotal to supporting cell growth, membrane function, and cell stress responses.^116^, ^117^, ^118^ PUFAs have been mechanistically linked to angiogenesis and thrombosis by modulating the function of endothelial cells and platelets,^55^ respectively, key cellular changes involved in PFVM. However, no study thus far has been conducted to examine the relationship between PFVM and LC-PUFA metabolism. Research in our group and others^59^^,^^119^ (GTEx etc.) have previously demonstrated that a group of SNPs in high linkage disequilibrium and located at the *FADS1-FADS2* locus are strong determinants for *FADS1*/*FADS2* expression in the liver and other tissues, with their minor alleles associated with lower FADS1/2 expression. Our study suggested that these alleles particularly reduce the expression of *FADS1* compared to *FADS2* and *FADS3* in the gene cluster region.^59^ Both *FADS1* and *FADS2* encode rate-limiting enzymes for PUFA desaturation while *FADS1*'s direct products are ARA and EPA (Fig. 1). Indeed, the SNPs at the locus are consistently associated with circulating LC-PUFA levels, e.g. the level of ARA, EPA and DHA in multiple large-scale genome-wide association studies (GWAS), including those performed in pregnant women.^68^^,^^70^^,^^120^, ^121^, ^122^, ^123^ Moreover, multi-omic studies also demonstrated that these polymorphisms determine the levels of metabolic derivatives of these LC-PUFA precursors.^95^ These metabolic derivatives are known to have broad biological functions. Both rs174538 and rs174556 are among the outstanding tagSNPs among these GWAS hits.^68^^,^^70^^,^^120^, ^121^, ^122^, ^123^ Moreover, these SNPs have been linked to other placental or perinatological health conditions.^68^^,^^124^ However, the role of these SNPs as well as the key gene *FADS1* regulated by these SNPs in PFVM have not been explored thus far.

Our data in this study showed that the low-function alleles of *FADS1* SNPs are associated with an increased risk of PFVM, particularly PFVM-SK. During pregnancy, foetal lipid metabolism, especially PUFA desaturation, remains immature, relying primarily on maternal PUFA supply. Therefore, maternal PUFA desaturation is a crucial factor in maintaining placental function, ensuring normal oxygen and nutrient supply and exchange for the foetus. Therefore, it is possible that PFVM pathogenesis could be attributed to the genetically reduced blood levels of LC-PUFAs in the maternal circulation system.

On the other hand, dietary PUFA levels can also influence the systemic availability of LC-PUFAs. It is known that the modern human diet is characterised by an oversupply of n-6 PUFAs including ARA while a low level of n-3 PUFAs. Previous studies have broadly observed that imbalanced n-3 vs n-6 PUFAs in modern human diet is associated with various placental and perinatological perturbations.^34^^,^^125^^,^^126^ Therefore, these low-function alleles may particularly reduce the endogenous bioproduction of n-3 LC-PUFAs, exacerbating the imbalanced n-3 vs n-6 PUFA supplies in modern diet. Indeed, our bioinformatic analysis suggested that PFVM-associated genes are also significantly related to ARA and its lipid derivatives. Interestingly, LC-PUFA biosynthesis is also subject to feedback regulation. Previous studies have shown that downstream LC-PUFAs, particularly DHA and, to a lesser extent, ARA, suppress Δ6-desaturase (*FADS2*) activity through negative feedback mechanisms to maintain lipid homoeostasis.^127^ Under conditions of impaired desaturase activity, such as in carriers of low-function *FADS1* alleles, direct dietary supplementation with downstream LC-PUFAs may help bypass reduced endogenous synthesis. We previously demonstrated that low-function allele carriers benefit more significantly from DHA supplementation for treating liver disease.^128^^,^^129^ Consistent with this concept, exogenous balanced supplementation of ARA and DHA largely rescued the functional defects induced by FADS1 inhibition in trophoblasts and endothelial cells in our study, suggesting that LC-PUFA supplementation, particularly DHA (given the sufficient dietary supply of ARA), may represent a potential nutritional strategy to improve placental vascular development in genetically susceptible pregnancies.^130^ In addition to reducing LC-PUFA availability, genetically reduced *FADS1* activity may also alter the production of downstream lipid mediators derived from ARA and EPA, including prostaglandins, leukotrienes, lipoxins, resolvins, and other eicosanoids and docosanoids.^95^ These bioactive mediators are key regulators for endothelial cell survival, angiogenesis, vascular remodelling, platelet activation, thrombosis, and inflammatory resolution at the maternal–foetal interface.^131^ Therefore, dysregulated production of these lipid mediators may represent an additional mechanism by which impaired PUFA desaturation contributes to PFVM. Although supplementation with ARA and DHA rescued the functional defects induced by FADS1 inhibition in trophoblasts and endothelial cells, whether these effects are mediated directly by LC-PUFAs or by their downstream lipid metabolites remains to be determined. Future studies should further examine the potential inter-relationships between dietary, circulatory, and placental levels of LC-PUFAs, as well as their lipids products and PFVM risk.

Notably, *FADS1* is ubiquitously expressed in human tissues.^119^ The genetic variants therefore should also possess impacts on cell and tissue-specific functions via altering the endogenous availability of LC-PUFAs. In this study, we analysed the transcriptomic profiles associated with the low-function alleles of the *FADS1* polymorphisms. Specifically, these alleles are associated with dysregulation of apoptotic signalling, enhanced cell stress response, as well as tissue development and repairs. These enriched pathways are largely consistent with those associated with PFVM or PFVM-SK. These shared pathways bridge the connection between FADS1 function and PFVM. Recent studies in both our lab^83^ and others have reported that low *FADS1* function is associated with elevated ER stress in cancer cells, immune cells, and others. Severe ER stress disrupts cell proliferation, halts the cell cycle, and induces cell death.^83^^,^^132^^,^^133^

Interestingly, our transcriptomic analysis highlighted the major involvement of trophoblast and endothelial cells as the profiles linked to the low-function alleles of *FADS1* polymorphisms. By using in vitro assays, we found that LC-PUFAs treatment and/or reduced *FADS1* activity influence cell death and limits tube formation function and growth factor release, migration or invasion, and even proliferation in endothelial cells and trophoblasts. Because placental endothelial cells form the foetal capillary network are responsible for maternal–foetal exchange, impaired endothelial migration, angiogenesis, and survival may not only disrupt placental vascular development but also reduce the efficiency of nutrient and oxygen transfer to the foetus by limiting placental perfusion and exchange capacity. Although nutrient transport was not directly evaluated in the present study, these findings suggest that trophoblast-endothelial dysfunction may represent an important mechanistic link between altered maternal lipid metabolism, PFVM, and impaired maternofetal nutrient exchange. Taken together, these findings suggest that, both the systemic regulation via modulating exogenous LC-PUFA levels and cell-specific *FADS1* function alteration, therefore modulating the endogenous level of LC-PUFAs, together contribute to the pathogenesis of PFVM, while exogenous supplementation of balanced level of LC-PUFAs can restore the impaired cell function due to the loss of *FADS1* function. Future studies should further explore the role of *FADS1* function at the maternal–foetal interface both in vitro and in vivo models.

This study highlights the clinical importance of monitoring pregnant women who exhibit low *FADS1* expression and reduced activity of PUFA desaturation. Should our findings be further validated, it is advisable that these individuals can be closely examined for blood vessel development and placental function throughout pregnancy. Additionally, supplementing downstream bioproducts of PUFA desaturation among the pregnant women carrying the *FADS1* low-function alleles may be beneficial in reducing the risk of vessel pathologies that can induce pregnancy complications. Given the imbalanced n-3 and n-6 PUFA and abundant level of ARA (n-6 PUFA) among the modern human diet, the reduced *FADS1* function owing to the genetic variants may particularly exacerbate the low level of EPA and DHA (both are n-3 PUFA) (Fig. 1). Currently, although dietary intake or supplementation of n-3 PUFAs are recommended by many guidelines in the US, e.g. the American Academy of Pediatrics and American College of Obstetricians and Gynaecologists, daily intake of long-chain n-3 PUFA i.e. EPA and DHA among over 95% pregnant women remains low.^134^ Our findings further support the necessity of n-3 LC-PUFA intake in pregnant women in general and highlights that those who carry the *FADS1* low-function alleles may particularly benefit from such an intake. However, pinpointing which specific LC-PUFA or their lipid product and a standard level that may exert the best outcome needs additional investigation and is out of the primary scope of the current study. Nevertheless, our data provide a rationale for future precision nutrition strategies targeting women at increased genetic risk of PFVM. From a research perspective, our study has explored placental molecular profiles underlying PFVM and identified several key genes and pathways potentially linked to the interplay between PUFA desaturation and placental pathologies. These elements provide new hypotheses for future investigation towards deciphering the complex mechanisms underlying these associations and developing targeted interventions to mitigate adverse pregnancy outcomes.

This study has several limitations that should be considered. First, as an observational study, causal relationships cannot be definitively established, and residual confounding by unmeasured factors, such as maternal diet, environmental exposures, medication use, or other metabolic conditions, cannot be completely excluded despite adjustment for key maternal covariates and the use of a causal modelling framework. One example is the maternal metabolic condition. While circulating LC-PUFAs is primarily processed in the liver, *FADS1* expression or function can be affected by various liver pathologies, e.g. hepatic steatosis.^59^^,^^128^ Fatty liver disease is highly prevalent, raising the possibility that maternal liver conditions may be a confounding factor for PFVM as well. In our cohort, however, data for maternal liver disease in our study cohort is not available. Future studies should consider these factors to further validate the findings of our current study. Second, this study failed to obtain foetal *FADS1* genotype, making it impossible to evaluate the impact of foetal genotype on PFVM. Previous studies have suggested that both maternal and foetal *FADS* genotypes may co-contribute to foetal blood level of LC-PUFAs, especially among foetal carriers of low-function allele homozygous genotype.^61^ Although we observed association between maternal genotype and PFVM, it is possible that foetal genotype may have additive or synergistic effect on the PFVM risk since compared to non-carriers, maternal low function-allele carriers are more likely to have foetuses carrying the low-function allele. Further examining this issue by obtaining clear foetal genotype e.g. via genotyping cord blood or infant samples is necessary. Third, our study also failed to quantify respective *FADS1* expression in maternal and foetal cells, missing the chance to directly evaluate the inter-relationship between maternal and foetal genotypes, *FADS1* expression among maternal and foetal cells, as well as PFVM. Signal-cell sequencing may be a valuable strategy to solve this problem. Fourth, our available samples (FFPE sections) do not allow us to profile the lipidomics in the placenta to further delineate the molecular mechanism underlying various genetic associations. We have started to collect fresh placenta tissue to further address this question in our continued investigation. Fifth, although our functional studies identified important roles for *FADS1* in trophoblasts and endothelial cells, its effects on other placental cell types, including immune cells and platelets, remain to be investigated. Unfortunately, there is currently a lack of good research models to recapitulate the complex and human-specific maternal–foetal interface. Future direction, e.g. placenta organoids^135^^,^^136^ may provide valuable tools for further investigation. Finally, there are some limitations in data analysis. For example, missing data in selected covariates required complete-case analyses; however, the consistency of effect estimates across adjusted and unadjusted models suggests that the primary findings are robust. In addition, some subgroup analyses were limited by small sample sizes, which may have introduced sparse-data bias^137^ and inflated effect size estimates, warranting cautious interpretation of the statistics. Also, enrichment analyses provide relative rankings rather than quantitative effect estimates with confidence intervals, and the in vitro functional studies were performed with a limited number of independent biological replicates. Lastly, our in-house IHC staining unfortunately did not produce sufficiently specific and reproducible positive staining in our placental sections. Therefore, *FADS1* protein localisation could not be reliably determined in our samples, and we instead referenced publicly available placental immunohistochemistry data from the Human Protein Atlas, as well as single-cell sequencing data to infer the cell types with *FADS1* expression. Future studies involving larger cohorts, various maternal and foetal tissue samples, more comprehensive exposure assessment, and validation in additional placental cell types will further clarify the role of *FADS1*-mediated fatty acid desaturation in placental vascular development and PFVM.

In summary, our results indicate that low maternal PUFA desaturation activity is associated with alterations in the transcriptomic profile (Graphical Abstract), with some of which contributing to the development of placental FVM. Concurrently, abnormal angiogenesis, migration, proliferation, and apoptosis in trophoblast and endothelial cells, triggered by low LC-PUFAs environment or *FADS1* deficiency, may mechanistically underly these foetal vessel pathologies. These findings underscore the critical role of *FADS1* and PUFA desaturation in maintaining placental health and highlight potential targets for therapeutic intervention to mitigate placental dysfunctions and their associated foetal vessel pathologies.

## Contributors

Conceptualisation: ZLiu, ZLong, CS, TM, DR, NP, JK, RF, and WL; Methodology: ZLiu, ZLong, SN, LT, RP, CS, ZP, YJ, TM, DR, NP, JK, RF, and WL; Validation: ZLiu, ZLong, SS, CS, TM, DR, NP, JK, RF, and WL; Formal analysis: ZLiu, CS, TM, DR, NP, JK, RF, and WL; Investigation: ZLiu, ZLong, SN, LT, SS, RP, CS, ZP, YJ, TM, DR, NP, JK, RF, and WL; Resources: CS, TM, DR, NP, JK, RF, and WL; Data curation: ZLiu, ZLong, SN, LT, SS, CS, TM, DR, NP, JK, RF, and WL; Writing- original draft: ZLiu, WL; Writing-review & editing: ZLiu, ZLong, SN, LT, RP, SS, CS, ZP, YJ, TM, DR, NP, JK, RF, and WL; Visualisation: ZLiu, WL; Supervision: WL; Project administration: ZLiu, WL; Funding acquisition: CS, TM, DR, NP, JK, RF, and WL. All authors read and approved the final version of the manuscript. Z. Liu and W. Liu accessed and verified the underlying data.

## Data sharing statement

All de-identified data and their detailed descriptions in this study as well as the analysis code used to generate the reported results are provided in the Supplementary Materials. Additional clinical, demographic, and laboratory testing data of the participants may be available upon reasonable request and applicable institutional approval.

## Declaration of interests

W.L. is currently a consultant of GeneToBe Inc., a co-founder of AlleleMed, Inc. and has a Patent application related to the application of degron-fused proteins (U.S. Patent Appl.: 64/119,695). However, these activities and the patent application are not related to this study. Other authors declare no competing interests.

## Appendix A Supplementary data

Supplementary Materials

Supplementary Database

Supplementary Western Blot

Supplementary Sharing Code

Supplementary Reagent Validation

Graphical Abstract **Graphical summary of *FADS1*-mediated PUFA desaturation and PFVM pathogenesis.** Low-function *FADS1* alleles were associated with reduced placental *FADS1* expression and increased PFVM risk, alongside changes in immune, apoptosis, and angiogenesis pathways; in vitro, LC-PUFA supplementation reversed the trophoblast and endothelial impairments caused by *FADS1* inhibition.
