# Supplementary material for: Decreased function of fatty acid desaturase 1 is associated with placental foetal vascular malperfusion and highlights the key role of the maternal–foetal interface
Source: eBioMedicine. 2026 Oct 17;133:106517. doi: 10.1016/j.ebiom.2026.106517 (PMC13696122; doi:10.1016/j.ebiom.2026.106517)
Supplement: Supplementary file 1 [file mmc1.docx]

**Supplemental figures and their legends**

**Supplemental Figure 1. SNPs are associated with *FADS1* gene expression in the human liver, placenta, and PFVM.** (A) Analysis from the GTEx database identified single nucleotide polymorphisms (SNPs) rs174538 and (B) rs174556 were significantly associated with *FADS1* gene expression in human livers (median and interquartile range; median values were labelled in the plots). P-values were obtained using a linear regression model analogous to the Matrix eQTL approach. (C-D) The dot graph indicated the association between *FADS1* SNP genotype and placental *FADS1* gene expression among pregnant women; (horizontal bars indicate mean values; n=74). (E) The dot graph indicated the association between placental *FADS1* gene expression and PFVM (horizontal bars indicate mean values; n=74). Statistical analysis was performed based on one-way ANOVA followed by *post-hoc* Tukey multiply comparisons or Welch’s t tests. All p values adjusted with the Tukey multiply comparisons or Welch’s t tests were labelled in the plots.

**Supplementary Figure 2. In-house evaluation of antibodies for immunohistochemistry (IHC) staining of FADS1 protein in liver and placental tissue.** (A) A representative human liver section stained using two commercially available FADS1 antibodies, Abcam ab126706 and Sigma-Aldrich HPA042705, respectively. Positive staining of FADS1 with the Sigma-Aldrich HPA042705 antibody can be observed. (B) Three representative human placental sections (10-15 samples for each antibody test) stained using two FADS1 antibodies. Neither antibody produced sufficiently consistent and reproducible FADS1-specific staining under the conditions tested in the placenta section, based on a careful review by the co-author Dr. Saab. These images are provided to document the antibody validation and quality-control assessment and were not included in subsequent biological or quantitative analyses.

**Supplemental Figure 3. Distribution of placental samples in this study.** (A) A total of 168 placental samples underwent cryosectioning for the evaluation of placental vessel pathologies as mentioned in (A). Among the diagnosed cases, approximately 67 pregnant women were identified with AVV, 30 with SK, 15 with HEV, 25 with FTHR, and 15 with CPTHR. (B) A total of 646 maternal blood samples were analyzed for FADS1 genetic information using genomic DNA (gDNA) analysis. Among these, 168 placental samples underwent cryosectioning for the evaluation of placental vessel pathologies. Additionally, 102 placental samples were used to generate libraries for transcriptomic analysis through RNA sequencing. A subset of 73 samples containing FADS1 genetic data, placental vessel pathology information, and transcriptomic profiles were selected to investigate the relationships among these factors.

**Supplemental Figure 4. Directed Acyclic Graph (DAG).** DAG showed the possible confounders for analysis between *FADS1* SNPs and PFVM, including maternal race, age, BMI, and GA.

**Supplemental Figure 5. Low functional alleles of maternal *FADS1* are associated with placental fetal vascular malperfusion** **(PFVM).** (A) Column bar graph showed the percentage of PFVM across different levels of *FADS1* genetic expression for rs174538 (B for rs174556; n=166). Statistical analysis was conducted using binary logistic regression (p value) and multiple logistic regression (adjust p value by excluding co-variates). (C) The PFVM, including Avascular Villi (AVV), Stromal Karyorrhexis (SK), Hemorrhagic Endovasculitis (HEV), Fetal Stem Mural Thrombosis (FTHR), and Chorionic Plate Vessel Mural Thrombosis (CPTHR), were scored by their immunohistology information. The heatmap illustrated the relationship between pathology scores and *FADS1* genetic expression level of rs174538 (D for rs174556). Statistical analysis was conducted using binary logistic regression (nominal p value on the left) and multiple logistic regression (adjust p value by excluding co-variates on the right).

**Supplemental Figure 6. FADS1 expression in different types of cells in the placenta.** (A-D) Singal-cell RNA sequencing data from different databases showed the top cell types which express the *FADS1* in the maternal and fetal interface of placenta (1-3). (E) The represented IHC images showed that the FADS1 expressed in the trophoblasts and endothelial cells in placenta. Data was directly cited from the Human Protein Atlas (4, 5). (<https://www.proteinatlas.org/ENSG00000149485-FADS1/tissue/placenta#img>)

**Supplemental Figure 7. LC-PUFAs induced mRNA expression of *FADS1* in trophoblast and endothelial cells.** The dot graph indicated the *FADS1* expression in the LC-PUFAs (equal concentration of ARA and DHA)-treated HUVEC and HTR-8 cells (n=4). Statistical analysis was performed based on Welch’s t tests. All nominal p values were labelled in the plots. All experiments were performed in 4 independent replicates. Horizontal bars indicated mean values.

**Supplemental Figure 8. Evaluation of the *FADS1* knockdown in HTR-8 cells.** (A) The Western blot images showed the expression of FADS1 of FADS1-knockdown (KD) cells in three independent replications. Vinculin was used as the housekeeping protein for loading control. (B) The dot graph showed the quantified relative FADS1 protein expression in the FADS1-KD (sh-FADS1) HTR-8 cells (horizontal bar indicates the mean; n=3). (C) The dot graph indicated the level of secreted growth factors (VEGF-A and PIGF) in the FADS1-KD (sh-FADS1) and control (sh-scramble) HTR-8 cells medium (horizontal bar indicates the mean; n=4). (D) Raw Western blot images for data presented in (A & B). Statistical analysis was performed based on Welch’s t tests. All Welch’s t tests p values were labelled in the plots. All experiments were performed in 3-4 independent replicates.

**Supplemental Tables**

| **Name** | **Target** | **Hairpin sequences** |
| --- | --- | --- |
| sh- *FADS1*  (SCBT; sc-96474-V) | FADS1 | GATCCGGATAGGTATGACCTATGTTTCAAGAGAACATAGGTCATACCTATCCTTTTT  GATCCCTCTAGGCATATTGATCATTTCAAGAGAATGATCAATATGCCTAGAGTTTTT  GATCCCCATGGAGAGGTTTGTCAATTCAAGAGATTGACAAACCTCTCCATGGTTTTT |

**Supplemental Table S1. Sequences for the shRNA experiment (Sequences are provided in 5’ to 3’ orientation).**

**Supplemental Table S2. Primer sequences for the RT-qPCR analysis (Sequences are provided in 5’ to 3’ orientation).**

| **Gene** | **Forward primer** | **Reverse primer** |
| --- | --- | --- |
| FADS1 | CAGAGCTGCCCTGACTCCTT | GCCTTCGCCGACATCATC |
| PPIA | AGGTCCCAAAGACAGCAGAA | GAAGTCACCACCCTGACACA |

**Supplemental Table S3. Confounder identification (univariate analysis) for the association between rs174538 and PFVM.**

| **Confounder** | **p_with_rs174538** | **p_with_PFVM** | **Meaning** |
| --- | --- | --- | --- |
| BMI | 0.23 | 0.48 | Not associated with X or Y → not a confounder |
| AGE | 0.14 | 0.025 | AGE affects PFVM but not the SNP strongly → possible confounder |
| RACE | 0.18 | 0.72 | Not associated → not a confounder |
| GA | 0.93 | 0.23 | Not associated with SNP → not a confounder |

| **Model** | **β** | **% Change** | **Interpretation** |
| --- | --- | --- | --- |
| Crude | -1.112 | — | baseline |
| Adjusted for BMI | -1.102 | 0.93% | negligible change |
| Adjusted for AGE | -1.064 | 4.37% | small change |
| Adjusted for RACE | -1.142 | –2.66% | negligible |
| Adjusted for GA | -1.136 | –2.11% | negligible |
| ALL covariates together | -1.117 | –0.42% | negligible |

**Supplemental Table S4. Confounder identification (multivariate analysis) for the association between rs174556 and PFVM.**

**.**

**Supplemental Table S5. Confounder identification (univariate analysis) for the association between rs174556 and PFVM.**

| **Confounder** | **p_with_rs174556** | **p_with_PFVM** | **Interpretation** |
| --- | --- | --- | --- |
| BMI | 0.395 | 0.473 | Not associated with SNP or PFVM → not a confounder |
| AGE | 0.325 | 0.024 | AGE is associated with PFVM but not genotype → possible but weak confounder |
| RACE | 0.057 | 0.669 | RACE is borderline related to genotype (p=0.0567) but not PFVM → not a confounder |
| GA | 0.647 | 0.258 | No association with SNP or PFVM → not a confounder |

**Supplemental Table S6. Confounder identification (multivariate analysis) for the association between rs174538 and PFVM.**

| **Model** | **β** | **% Change** | **Interpretation** |
| --- | --- | --- | --- |
| Crude | –0.6758 | — | Baseline SNP–PFVM association |
| Adjusted for BMI | –0.6663 | 1.41% | No meaningful change |
| Adjusted for AGE | –0.6381 | 5.58% | Small change, still < 10% |
| Adjusted for RACE | –0.7157 | –5.90% | Small negative change |
| Adjusted for GA | –0.7030 | –4.02% | Very small change |
| Adjusted for ALL | –0.7216 | –6.78% | Combined change still < 10% |

**Supplemental Table S7. Top enriched metabolites associated with PFVM-DEGs.**

| **Index** | **Term** | **P-value** | **Odds Ratio** | **Combined Score** |
| --- | --- | --- | --- | --- |
| 1 | Linoleic acid (HMDB00673) | 8.94E-04 | 17.53412 | 123.0781 |
| 2 | Arachidonic acid (HMDB01043) | 0.001557 | 14.30708 | 92.49825 |
| 3 | 19-Hydroxyandrost-4-ene-3,17-dione (HMDB03955) | 0.00456 | 22.02769 | 118.7381 |
| 4 | C19H28O3 (HMDB03956) | 0.00456 | 22.02769 | 118.7381 |
| 5 | 6-Hydroxymelatonin (HMDB04081) | 0.00456 | 22.02769 | 118.7381 |
| 6 | 16(R)-HETE (HMDB04680) | 0.00456 | 22.02769 | 118.7381 |
| 7 | 11,12,15-THETA (HMDB04684) | 0.00456 | 22.02769 | 118.7381 |
| 8 | (5Z,8Z,12E)-14,15-Epoxy-11-hydroxyeicosa-5,8,12-trienoic acid (HMDB04693) | 0.00456 | 22.02769 | 118.7381 |
| 9 | (5Z,8Z,12E)-11,14,15-Trihydroxyeicosa-5,8,12-trienoic acid (HMDB04694) | 0.00456 | 22.02769 | 118.7381 |
| 10 | 9,10-Epoxyoctadecenoic acid (HMDB04701) | 0.00456 | 22.02769 | 118.7381 |
| 11 | 12,13-EpOME (HMDB04702) | 0.00456 | 22.02769 | 118.7381 |
| 12 | (5Z,8Z,13E)-(15S)-11,12-Epoxy-15-hydroxyeicosa-5,8,13-trienoic acid (HMDB05050) | 0.00456 | 22.02769 | 118.7381 |
| 13 | SMR000538886 (HMDB06768) | 0.00456 | 22.02769 | 118.7381 |
| 14 | C19H28O3 (HMDB06769) | 0.00456 | 22.02769 | 118.7381 |
| 15 | 19S-hydroxy-5Z,8Z,11Z,14Z-eicosatetraenoic acid (HMDB11136) | 0.00456 | 22.02769 | 118.7381 |
| 16 | Valproic acid (HMDB01877) | 0.004961 | 21.02537 | 111.5657 |
| 17 | 5,6-Epoxy-8,11,14-eicosatrienoic acid (HMDB02190) | 0.004961 | 21.02537 | 111.5657 |
| 18 | 8,9-EET (HMDB02232) | 0.004961 | 21.02537 | 111.5657 |
| 19 | C19H28O2 (HMDB03959) | 0.004961 | 21.02537 | 111.5657 |
| 20 | 14,15-Epoxy-5,8,11-eicosatrienoic acid (HMDB04264) | 0.004961 | 21.02537 | 111.5657 |
| 21 | 11,12-EET (HMDB04673) | 0.004961 | 21.02537 | 111.5657 |
| 22 | Parathion (HMDB01355) | 0.005377 | 20.11021 | 105.0902 |
| 23 | Hydrogen sulfide (HMDB03276) | 0.005377 | 20.11021 | 105.0902 |
| 24 | Simvastatin (HMDB05007) | 0.005377 | 20.11021 | 105.0902 |
| 25 | Caffeine (HMDB01847) | 0.005808 | 19.27132 | 99.21871 |
| 26 | Paraxanthine (HMDB01860) | 0.005808 | 19.27132 | 99.21871 |
| 27 | Citalopram (HMDB05038) | 0.005808 | 19.27132 | 99.21871 |
| 28 | C34H34N4O4.Fe (HMDB03178) | 0.006616 | 5.69899 | 28.5989 |
| 29 | NADPH (HMDB00221) | 0.00732 | 5.529972 | 27.19143 |
| 30 | Formic acid (HMDB00142) | 0.008714 | 15.4124 | 73.0986 |
| 31 | (5Z,8Z,11Z,13E)-(15S)-15-Hydroperoxyicosa-5,8,11,13-tetraenoate (HMDB04244) | 0.008714 | 15.4124 | 73.0986 |
| 32 | Melatonin (HMDB01389) | 0.0098 | 14.44767 | 66.82601 |
| 33 | Formaldehyde (HMDB01426) | 0.010364 | 14.00916 | 64.01321 |
| 34 | Androstenedione (HMDB00053) | 0.010943 | 13.59644 | 61.38881 |
| 35 | Oxoglutaric acid (HMDB00208) | 0.012142 | 12.83979 | 56.63766 |
| 36 | Codeine (HMDB04995) | 0.015377 | 11.27113 | 47.05599 |
| 37 | 1-hexadecyl-2-(9Z,12Z-octadecadienoyl)-sn-glycero-3-phosphocholine (HMDB11151) | 0.015377 | 11.27113 | 47.05599 |
| 38 | PC(20:3(8Z,11Z,14Z)/20:1(11Z)) (HMDB11201) | 0.015377 | 11.27113 | 47.05599 |
| 39 | PC(22:1(13Z)/18:1(11Z)) (HMDB11202) | 0.015377 | 11.27113 | 47.05599 |
| 40 | PC(P-16:0/14:0) (HMDB11203) | 0.015377 | 11.27113 | 47.05599 |

**Supplemental Table S8. Top cell types enriched based on the enrichment analysis of PFVM-DEGs.**

| **Index** | **Term** | **P-value** | **Odds Ratio** | **Combined Score** |
| --- | --- | --- | --- | --- |
| 1 | Fetal Development-Placenta-Vascular Endothelial Cells | 0.038918 | 28.5977 | 92.83698 |
| 2 | Fetal Development-Adrenal-Vascular Endothelial Cells | 0.038918 | 28.5977 | 92.83698 |
| 3 | Fetal Development-Muscle-Vascular Endothelial Cells | 0.038918 | 28.5977 | 92.83698 |
| 4 | Fetal Development-Hepatoblasts | 0.038918 | 28.5977 | 92.83698 |
| 5 | Fetal Development-Intestinal Epithelial Cells | 0.038918 | 28.5977 | 92.83698 |
| 6 | Fetal Development-Epicardial Fat Cells | 0.038918 | 28.5977 | 92.83698 |
| 7 | Fetal Development-Parietal And Chief Cells | 0.038918 | 28.5977 | 92.83698 |
| 8 | Fetal Development-CSH1 CSH2 Positive Cells | 0.038918 | 28.5977 | 92.83698 |
| 9 | Fetal Development-CCL19 CCL21 Positive Cells | 0.038918 | 28.5977 | 92.83698 |
| 10 | Fetal Development-Lung-Stromal Cells | 0.038918 | 28.5977 | 92.83698 |
| 11 | Fetal Development-Pancreas-Stromal Cells | 0.038918 | 28.5977 | 92.83698 |
| 12 | Fetal Development-Pancreas-Lymphatic Endothelial Cells | 0.038918 | 28.5977 | 92.83698 |
| 13 | Fetal Development-Muscle-Lymphatic Endothelial Cells | 0.038918 | 28.5977 | 92.83698 |
| 14 | Fetal Development-Heart-Lymphatic Endothelial Cells | 0.038918 | 28.5977 | 92.83698 |
| 15 | Fetal Development-Spleen-Myeloid Cells | 0.038918 | 28.5977 | 92.83698 |
| 16 | Fetal Development-Spleen-STC2 TLX1 Positive Cells | 0.038918 | 28.5977 | 92.83698 |
| 17 | Fetal Development-Pancreas-Smooth Muscle Cells | 0.038918 | 28.5977 | 92.83698 |
| 18 | Fetal Development-Stomach-MUC13 DMBT1 Positive Cells | 0.038918 | 28.5977 | 92.83698 |
| 19 | Fetal Development-Heart-Epicardial Fat Cells | 0.038918 | 28.5977 | 92.83698 |
| 20 | Fetal Development-Adrenal-CSH1 CSH2 Positive Cells | 0.038918 | 28.5977 | 92.83698 |
| 21 | Fetal Development-Pancreas-CCL19 CCL21 Positive Cells | 0.038918 | 28.5977 | 92.83698 |
| 22 | Fetal Development-Stromal Cells | 0.038918 | 28.5977 | 92.83698 |
| 23 | Fetal Development-Lymphatic Endothelial Cells | 0.038918 | 28.5977 | 92.83698 |
| 24 | Fetal Development-STC2 TLX1 Positive Cells | 0.038918 | 28.5977 | 92.83698 |

**Supplemental Table S9. Top cell types enriched based on the enrichment analysis of PFVM SK-DEGs.**

| **Index** | **Term** | **P-value** | **Odds Ratio** | **Combined Score** |
| --- | --- | --- | --- | --- |
| 1 | Fetal Development-Placenta-IGFBP1 DKK1 Positive Cells | 0.030631 | 36.62316 | 127.6592 |
| 2 | Fetal Development-Cerebrum-Vascular Endothelial Cells | 0.030631 | 36.62316 | 127.6592 |
| 3 | Fetal Development-Lymphatic Endothelial Cells | 0.030631 | 36.62316 | 127.6592 |
| 4 | Fetal Development-Thymus-Antigen Presenting Cells | 0.030631 | 36.62316 | 127.6592 |
| 5 | Fetal Development-Antigen Presenting Cells | 0.030631 | 36.62316 | 127.6592 |
| 6 | Fetal Development-Stomach-MUC13 DMBT1 Positive Cells | 0.030631 | 36.62316 | 127.6592 |

**Supplemental Table S10. Top cell types enriched based on the enrichment analysis of DEGs associated with both rs174538 & rs174556s.**

| **Index** | **Term** | **P-value** | **Odds Ratio** | **Combined Score** |
| --- | --- | --- | --- | --- |
| 1 | Fetal Development-Stomach-Lymphatic Endothelial Cells | 0.021397879 | 53.04255319 | 203.9201587 |
| 2 | Fetal Development-Adrenal-Stromal Cells | 0.021397879 | 53.04255319 | 203.9201587 |
| 3 | Fetal Development-Adrenal-Adrenocortical Cells | 0.021397879 | 53.04255319 | 203.9201587 |
| 4 | Fetal Development-Eye-Bipolar Cells | 0.021397879 | 53.04255319 | 203.9201587 |
| 5 | Fetal Development-Adrenal-Lymphoid Cells | 0.021397879 | 53.04255319 | 203.9201587 |
| 6 | Fetal Development-Adrenal-Myeloid Cells | 0.021397879 | 53.04255319 | 203.9201587 |
| 7 | Fetal Development-Bipolar Cells | 0.021397879 | 53.04255319 | 203.9201587 |
| 8 | Fetal Development-Adrenocortical Cells | 0.021397879 | 53.04255319 | 203.9201587 |
| 9 | Fetal Development-Adrenal-Schwann Cells | 0.021397879 | 53.04255319 | 203.9201587 |
| 10 | Fetal Development-Adrenal-Megakaryocytes | 0.021397879 | 53.04255319 | 203.9201587 |
| 11 | Fetal Development-Photoreceptor Cells | 0.021397879 | 53.04255319 | 203.9201587 |
| 12 | Fetal Development-Eye-Photoreceptor Cells | 0.021397879 | 53.04255319 | 203.9201587 |
| 13 | Fetal Development-Spleen-Stromal Cells | 0.021397879 | 53.04255319 | 203.9201587 |

**References**

1.	Suryawanshi H, Morozov P, Straus A, Sahasrabudhe N, Max KEA, Garzia A, et al. A single-cell survey of the human first-trimester placenta and decidua. Sci Adv. 2018;4(10):eaau4788.

2.	Vento-Tormo R, Efremova M, Botting RA, Turco MY, Vento-Tormo M, Meyer KB, et al. Single-cell reconstruction of the early maternal-fetal interface in humans. Nature. 2018;563(7731):347-53.

3.	Arutyunyan A, Roberts K, Troule K, Wong FCK, Sheridan MA, Kats I, et al. Spatial multiomics map of trophoblast development in early pregnancy. Nature. 2023;616(7955):143-51.

4.	Uhlen M, Fagerberg L, Hallstrom BM, Lindskog C, Oksvold P, Mardinoglu A, et al. Proteomics. Tissue-based map of the human proteome. Science. 2015;347(6220):1260419.

5.	Karlsson M, Zhang C, Mear L, Zhong W, Digre A, Katona B, et al. A single-cell type transcriptomics map of human tissues. Sci Adv. 2021;7(31).
