# Supplementary material for: Decreased function of fatty acid desaturase 1 is associated with placental foetal vascular malperfusion and highlights the key role of the maternal–foetal interface
Source: eBioMedicine. 2026 Oct 17;133:106517. doi: 10.1016/j.ebiom.2026.106517 (PMC13696122; doi:10.1016/j.ebiom.2026.106517)
Supplement: Supplementary file 3 [file mmc4.docx]

***Binary logistic regression***

\# Load necessary libraries

library(tidyverse)

library(readxl)

library(openxlsx)

\# Read data from Excel file

DATA <- read_excel("C:/Users/fz5707/SNP1.xlsx")

\# Make sure rs174538 is treated as an ordinal predictor (factor with order)

\# If you want it treated as numeric, you can skip this.

DATA$rs174538 <- factor(DATA$rs174538, levels = c(0, 1, 2), ordered = TRUE)

\# Initialize an empty data frame to store all results

All_Results <- data.frame()

\# Loop through the first 25 variables as dependent variables (Y, binary 0/1)

for (i in 33:38) {

dep_name <- colnames(DATA)[i]

\# Skip if this column is 'rs174538'

if (dep_name == "rs174538") next

\# Ensure Y is binary factor (0/1)

\# (If it's already 0/1 numeric, glm will still work, but factor is safer.)

y_vec <- DATA[[i]]

\# Skip if column is all NA or has no variation (all 0 or all 1)

if (all(is.na(y_vec)) || length(na.omit(unique(y_vec))) < 2) next

DATA$Y_bin <- as.factor(y_vec)

\# Build logistic regression formula: Y_bin ~ rs174538

formula <- as.formula("Y_bin ~ rs174538")

\# Fit logistic regression

glm_result <- glm(formula, data = DATA, family = binomial)

summary_result <- summary(glm_result)

\# Extract coefficients table

coef_table <- summary_result$coefficients

\# We want the overall rs174538 effect:

\# If rs174538 is a factor with 3 levels, you'll get contrasts like:

\#	rs174538.L, rs174538.Q	(orthogonal polynomial contrasts)

\# or for dummy coding, rs1745381, rs1745382, etc.

\# Here I assume default contrasts for ordered factor: ".L" and ".Q".

\# If you prefer treat rs174538 as numeric, set it numeric above and use "rs174538".

\# Case 1: rs174538 numeric predictor

\# (uncomment this block if you made rs174538 numeric instead of factor)

\# if ("rs174538" %in% rownames(coef_table)) {

\#	coll_row <- coef_table["rs174538", ]

\# } else {

\#	next

\# }

\# Case 2: rs174538 as ordered factor with polynomial contrasts (default)

\# We collect all rows that start with "rs174538"

coll_rows <- grep("^rs174538", rownames(coef_table), value = TRUE)

if (length(coll_rows) == 0) next

\# For simplicity, just take the first contrast (often the linear trend)

coll_name <- coll_rows[1]

coll_row <- coef_table[coll_name, ]

\# Pseudo R² (McFadden-like): 1 - deviance/null.deviance

pseudo_R2 <- 1 - (glm_result$deviance / glm_result$null.deviance)

\# Add this result as one row into the big table

All_Results <- rbind(

All_Results,

data.frame(

Dependent_Variable = dep_name,

Predictor	= coll_name,

Coefficient	= coll_row["Estimate"],

Std_Error	= coll_row["Std. Error"],

Z_value	= coll_row["z value"],

P_value	= coll_row["Pr(>|z|)"],

Pseudo_R2	= pseudo_R2

)

)

}

\# Write everything into ONE Excel sheet

write.xlsx(

All_Results,

file	= "C:/Users/FZ5707/rs174538binaryY.xlsx",

sheetName = "rs174538_Logistic_Regression",

rowNames = FALSE

)

***Multiply binary logistic regression***

\# =========================

\# Adjusted binary logistic regression:

\# PFVM (0/1) ~ each rs174538 + BMI + AGE + RACE + GA

\# + multiple comparison correction (FDR/BH) across rs174538s

\# =========================

library(tidyverse)

library(readxl)

library(openxlsx)

\# ---- Read data ----

DATA <- read_excel("C:/Users/fz5707/SNP.xlsx")

\# ---- Force PFVM to numeric and keep only 0/1 ----

DATA <- DATA %>%

mutate(

PFVM_num = suppressWarnings(as.numeric(as.character(PFVM))),

PFVM_num = ifelse(PFVM_num %in% c(0, 1), PFVM_num, NA_real_),

PFVM = factor(PFVM_num, levels = c(0, 1)),

\# Make covariates numeric safely (in case they are character/factor)

AGE = suppressWarnings(as.numeric(as.character(AGE))),

BMI = suppressWarnings(as.numeric(as.character(BMI))),

RACE = suppressWarnings(as.numeric(as.character(RACE))),

GA	= suppressWarnings(as.numeric(as.character(GA)))

)

\# ---- rs174538 columns ----

rs174538_cols <- colnames(DATA)[75:185]

\# ---- Results holder ----

All_Results <- data.frame()

for (rs174538 in rs174538_cols) {

\# robust numeric conversion for rs174538 (handles factor/character safely)

x_raw <- DATA[[rs174538]]

x_num <- suppressWarnings(as.numeric(as.character(x_raw)))

df <- DATA %>%

transmute(

PFVM = PFVM,

rs174538 = x_num,

AGE = AGE,

BMI = BMI,

RACE = RACE,

GA	= GA

) %>%

filter(

!is.na(PFVM),

!is.na(rs174538),

!is.na(AGE),

!is.na(BMI),

!is.na(RACE),

!is.na(GA)

)

\# Skip unusable rs174538 variables

if (nrow(df) < 10) next

if (length(unique(df$PFVM)) < 2) next

if (length(unique(df$rs174538)) < 2) next

\# ---- Fit adjusted logistic regression ----

fit <- tryCatch(

glm(PFVM ~ rs174538 + AGE + BMI + RACE + GA, data = df, family = binomial),

error = function(e) NULL

)

if (is.null(fit)) next

sm <- summary(fit)$coefficients

if (!("rs174538" %in% rownames(sm))) next

beta <- sm["rs174538", "Estimate"]

se	<- sm["rs174538", "Std. Error"]

zval <- sm["rs174538", "z value"]

pval <- sm["rs174538", "Pr(>|z|)"]

\# Odds ratio + 95% CI for rs174538 (adjusted)

OR	<- exp(beta)

OR_L <- exp(beta - 1.96 * se)

OR_U <- exp(beta + 1.96 * se)

All_Results <- rbind(

All_Results,

data.frame(

rs174538 = rs174538,

Beta = beta,

SE = se,

Z = zval,

P = pval,

OR = OR,

OR_L = OR_L,

OR_U = OR_U,

N = nrow(df),

stringsAsFactors = FALSE

)

)

}

\# ---- Multiple testing correction across rs174538s ----

if (nrow(All_Results) > 0) {

All_Results$P_adj_FDR <- p.adjust(All_Results$P, method = "BH")

All_Results <- All_Results %>% arrange(P_adj_FDR, P)

}

\# ---- Export ----

write.xlsx(

All_Results,

file = "C:/Users/FZ5707/PFVM_Adjusted_Logistic_rs174538s_FDR.xlsx",

sheetName = "Adj_Logistic_PFVM",

rowNames = FALSE

)

***Multiply linear regression***

\# =========================

\# Multiple linear regression for placenta metrics (log2) ~ PFVM (log2) + covariates

\# Y = columns 3:73

\# X = PFVM

\# Covariates = AGE, BMI, RACE, GA

\# =========================

library(tidyverse)

library(readxl)

library(openxlsx)

\# ---- Read data ----

DATA <- read_excel("C:/Users/fz5707/SNP.xlsx")

\# ---- Predictor + covariates ----

x_var <- "PFVM"

covars <- c("AGE", "BMI", "RACE", "GA")

\# ---- Y columns (placenta condition metrics) ----

y_cols <- colnames(DATA)[1:2559]

\# ---- Results holder ----

All_Results <- data.frame()

\# ---- Loop through Y outcomes ----

for (y_name in y_cols) {

y <- DATA[[y_name]]

x <- DATA[[x_var]]

\# Skip unusable variables

if (all(is.na(y)) || length(unique(na.omit(y))) < 2) next

if (all(is.na(x)) || length(unique(na.omit(x))) < 2) next

\# Build analysis dataframe

df <- DATA %>%

transmute(

Y	= as.numeric(y),

X	= as.numeric(.data[[x_var]]),

AGE = as.numeric(.data[["AGE"]]),

BMI = as.numeric(.data[["BMI"]]),

RACE = as.numeric(.data[["RACE"]]),

GA = as.numeric(.data[["GA"]])

) %>%

filter(

!is.na(Y), !is.na(X),

!is.na(AGE), !is.na(BMI), !is.na(RACE), !is.na(GA)

)

\# Need enough samples (at least a bit more conservative for multivariable model)

if (nrow(df) < 20) next

\# ---- Fit multiple linear regression ----

fit <- tryCatch(

lm(Y ~ X + AGE + BMI + RACE + GA, data = df),

error = function(e) NULL

)

if (is.null(fit)) next

sm <- summary(fit)

coef_tab <- sm$coefficients

\# Extract X (PFVM) results

beta <- coef_tab["X", "Estimate"]

se	<- coef_tab["X", "Std. Error"]

tval <- coef_tab["X", "t value"]

pval <- coef_tab["X", "Pr(>|t|)"]

\# 95% CI for beta (PFVM)

ci <- tryCatch(confint(fit, level = 0.95), error = function(e) NULL)

if (is.null(ci)) next

beta_L <- ci["X", 1]

beta_U <- ci["X", 2]

\# Model fit metrics

r2	<- sm$r.squared

adjr2 <- sm$adj.r.squared

All_Results <- rbind(

All_Results,

data.frame(

Outcome = y_name,

Beta_PFVM = beta,

SE_PFVM = se,

T_PFVM = tval,

P_PFVM = pval,

Beta_L95 = beta_L,

Beta_U95 = beta_U,

R2 = r2,

Adj_R2 = adjr2,

N = nrow(df),

stringsAsFactors = FALSE

)

)

}

\# ---- Multiple testing correction ----

All_Results$P_adj_FDR <- p.adjust(All_Results$P_PFVM, method = "BH")

\# Sort by adjusted p

All_Results <- All_Results %>% arrange(P_adj_FDR, P_PFVM)

\# ---- Export ----

write.xlsx(

All_Results,

file = "C:/Users/FZ5707/PlacentaMetrics_MultipleLinearRegression_PFVM_Adjusted.xlsx",

sheetName = "MLR_Y_vs_PFVM_adj",

rowNames = FALSE

)

***Confounder***

#############################################

\# Confounder analysis for rs174556 & PFVM

\# Confounders tested: BMI, AGE, RACE, GA

#############################################

\# Load libraries

library(tidyverse)

library(readxl)

library(broom)

library(openxlsx)

\# Read your Excel file

DATA <- read_excel("C:/Users/fz5707/SNP.xlsx")

\# Ensure correct data types

DATA <- DATA %>%

mutate(

PFVM	= as.numeric(PFVM), # binary 0/1

rs174556 = as.numeric(rs174556), # genotype

BMI	= as.numeric(BMI),

AGE	= as.numeric(AGE),

GA	= as.numeric(GA),

RACE	= as.numeric(RACE)

)

\# Keep only complete cases

vars_needed <- c("PFVM", "rs174556", "BMI", "AGE", "GA", "RACE")

DATA_cc <- DATA %>% drop_na(all_of(vars_needed))

###############################################################

\# PART 1: Check associations (Confounder → X and Confounder → Y)

###############################################################

covariates <- c("BMI", "AGE", "RACE", "GA")

Assoc_Results <- data.frame(

Confounder = covariates,

p_with_rs174556 = NA_real_,

p_with_PFVM	= NA_real_

)

for (i in seq_along(covariates)) {

covar <- covariates[i]

\# Association with X = rs174556

model_X <- lm(as.formula(paste("rs174556 ~", covar)), data = DATA_cc)

tidy_X <- tidy(model_X)

p_X	<- tidy_X$p.value[tidy_X$term == covar]

\# Association with Y = PFVM (logistic regression)

model_Y <- glm(as.formula(paste("PFVM ~", covar)),

data = DATA_cc, family = binomial)

tidy_Y <- tidy(model_Y)

p_Y	<- tidy_Y$p.value[tidy_Y$term == covar]

Assoc_Results$p_with_rs174556[i] <- ifelse(length(p_X)>0, p_X, NA)

Assoc_Results$p_with_PFVM[i]	<- ifelse(length(p_Y)>0, p_Y, NA)

}

###############################################################

\# PART 2: Change-in-estimate confounder analysis

###############################################################

\# Crude model

model_crude <- glm(PFVM ~ rs174556, data = DATA_cc, family = binomial)

tidy_crude <- tidy(model_crude)

beta_crude <- tidy_crude$estimate[tidy_crude$term == "rs174556"]

se_crude	<- tidy_crude$std.error[tidy_crude$term == "rs174556"]

p_crude	<- tidy_crude$p.value[tidy_crude$term == "rs174556"]

OR_crude	<- exp(beta_crude)

CI_crude	<- exp(beta_crude + c(-1,1)*1.96*se_crude)

Confounder_Table <- data.frame(

Model	= "Crude (no covariates)",

Added_Confounder	= "None",

Beta_rs174556	= beta_crude,

OR_rs174556	= OR_crude,

OR_Lower95CI	= CI_crude[1],

OR_Upper95CI	= CI_crude[2],

Pvalue_rs174556	= p_crude,

Percent_Change_Beta = NA_real_

)

\# Adjust one-by-one

for (covar in covariates) {

model_adj <- glm(as.formula(

paste("PFVM ~ rs174556 +", covar)),

data = DATA_cc, family = binomial

)

tidy_adj <- tidy(model_adj)

beta_adj <- tidy_adj$estimate[tidy_adj$term == "rs174556"]

se_adj	<- tidy_adj$std.error[tidy_adj$term == "rs174556"]

p_adj	<- tidy_adj$p.value[tidy_adj$term == "rs174556"]

OR_adj <- exp(beta_adj)

CI_adj <- exp(beta_adj + c(-1,1)*1.96*se_adj)

pct_change <- 100 * (beta_adj - beta_crude) / abs(beta_crude)

Confounder_Table <- rbind(

Confounder_Table,

data.frame(

Model	= paste("Adjusted for", covar),

Added_Confounder	= covar,

Beta_rs174556	= beta_adj,

OR_rs174556	= OR_adj,

OR_Lower95CI	= CI_adj[1],

OR_Upper95CI	= CI_adj[2],

Pvalue_rs174556	= p_adj,

Percent_Change_Beta = pct_change

)

)

}

\# Full model with all covariates

model_full <- glm(PFVM ~ rs174556 + BMI + AGE + GA + RACE,

data = DATA_cc, family = binomial)

tidy_full <- tidy(model_full)

beta_full <- tidy_full$estimate[tidy_full$term == "rs174556"]

se_full	<- tidy_full$std.error[tidy_full$term == "rs174556"]

p_full	<- tidy_full$p.value[tidy_full$term == "rs174556"]

OR_full <- exp(beta_full)

CI_full <- exp(beta_full + c(-1,1)*1.96*se_full)

pct_change_full <- 100 * (beta_full - beta_crude) / abs(beta_crude)

Confounder_Table <- rbind(

Confounder_Table,

data.frame(

Model	= "Adjusted for ALL (BMI + AGE + GA + RACE)",

Added_Confounder	= "All",

Beta_rs174556	= beta_full,

OR_rs174556	= OR_full,

OR_Lower95CI	= CI_full[1],

OR_Upper95CI	= CI_full[2],

Pvalue_rs174556	= p_full,

Percent_Change_Beta = pct_change_full

)

)

###############################################################

\# PART 3: Export both tables into ONE Excel file

###############################################################

output_path <- "C:/Users/fz5707/Confounder_Results_rs174556_PFVM.xlsx"

wb <- createWorkbook()

addWorksheet(wb, "Assoc_X_Y")

writeData(wb, "Assoc_X_Y", Assoc_Results)

addWorksheet(wb, "Change_in_Estimate")

writeData(wb, "Change_in_Estimate", Confounder_Table)

saveWorkbook(wb, output_path, overwrite = TRUE)

message("Excel export complete: ", output_path)
