# Supplementary material for: Decreased function of fatty acid desaturase 1 is associated with placental foetal vascular malperfusion and highlights the key role of the maternal–foetal interface
Source: eBioMedicine. 2026 Oct 17;133:106517. doi: 10.1016/j.ebiom.2026.106517 (PMC13696122; doi:10.1016/j.ebiom.2026.106517)
Supplement: Supplementary file 4 [file mmc5.docx]

**Reagent Validation**

**HTR-8/SVneo**

The human first-trimester extravillous trophoblast cell line HTR-8/SVneo was obtained from the American Type Culture Collection (ATCC; CRL-3271; RRID: CVCL_7162; Manassas, VA, USA). Cells were maintained according to the supplier’s recommendations. Cell morphology was routinely monitored throughout the study, and cultures were periodically tested and confirmed to be free of mycoplasma contamination using PCR-based detection. Experiments were performed using low-passage cultures after thawing from authenticated frozen stocks.

**Human umbilical vein endothelial cells (HUVECs)**

Primary HUVECs were obtained from Lonza (Basel, Switzerland) and Thermo Fisher Scientific (Waltham, MA, USA), as described in the corresponding Methods section. Both suppliers provide donor-derived primary endothelial cells that undergo quality control prior to release, including assessment of endothelial morphology, viability, and endothelial cell identity. Cells were cultured according to the manufacturers’ protocols and used at early passages. Cultures were routinely examined for morphology and confirmed to be free of mycoplasma contamination.

**FADS1 antibody for Western blotting**

The anti-FADS1 antibody used for Western blotting (Abcam, ab126706; RRID: AB_11130088) was previously validated in our study (1). Specifically, shRNA-mediated FADS1 depletion resulted in a marked reduction in the corresponding FADS1 protein signal by Western blotting, providing experimental evidence of antibody specificity. In the present study, the antibody detected a band at the expected molecular weight, further supporting its suitability for detecting human FADS1.

**Vinculin and secondary antibodies**

Vinculin was used as the loading control for Western blotting and was detected using the Vinculin (E1E9V) Rabbit Monoclonal Antibody (Cell Signaling Technology, Danvers, MA, USA; catalog no. 13901; RRID: AB_2728768). This antibody is validated by the manufacturer for Western blotting and recognizes endogenous total Vinculin. An HRP-linked anti-rabbit IgG secondary antibody (Cell Signaling Technology; catalog no. 7074; RRID: AB_2099233) was used for chemiluminescent detection of the rabbit primary antibodies according to the manufacturer’s recommendations.

**Attempted FADS1 antibody validation for immunohistochemistry**

Two commercially available FADS1 antibodies, Abcam ab126706 and Sigma-Aldrich HPA042705, were evaluated for in-house immunohistochemical (IHC) staining of human placental tissues. However, neither antibody produced sufficiently consistent and reproducible staining under the experimental conditions tested (**Supplemental Figure 2**). Therefore, the in-house FADS1 IHC validation was discontinued, and no IHC staining data generated using either antibody are presented in the main manuscript. The placental FADS1 IHC images presented in the manuscript were obtained from the publicly available Human Protein Atlas and are included only as external reference data.

**General quality control**

All cell cultures were maintained under sterile conditions and routinely monitored for contamination and morphology. Experiments were performed using actively growing cells within the recommended passage ranges. Antibodies and other reagents were stored, handled, and used according to the manufacturers’ instructions.

**Reference**

1. Heravi G, Liu Z, Herroon M, Wilson A, Fan YY, Jiang Y, et al. Targeting polyunsaturated fatty acids desaturase FADS1 inhibits renal cancer growth via ATF3-mediated ER stress response. *Biomed Pharmacother.* 2025;186:118006.
