# Supplementary material for: Decreased function of fatty acid desaturase 1 is associated with placental foetal vascular malperfusion and highlights the key role of the maternal–foetal interface
Source: eBioMedicine. 2026 Oct 17;133:106517. doi: 10.1016/j.ebiom.2026.106517 (PMC13696122; doi:10.1016/j.ebiom.2026.106517)
Supplement: Supplementary file 5 [file mmc3.docx]

**Uncropped full blots with clear labelling and controls**
